# Disinformation as an obstructionist strategy in climate change mitigation: a review of the scientific literature for a systemic understanding of the phenomenon

**DOI:** 10.12688/openreseurope.18180.1

**Published:** 2024-08-06

**Authors:** Manuel Gertrudix, Alejandro Carbonell-Alcocer, Rubén Arcos, Cristina M. Arribas, Valeri Codesido-Linares, Nerea Benítez-Aranda

**Affiliations:** 1 Grupo Ciberimaginario, XR COM LAB, Faculty of Communication Sciences, Universidad Rey Juan Carlos (ROR 01v5cv687), Madrid, Community of Madrid, 28943, Spain

**Keywords:** Climate Disinformation, Obstructionism, Misinformation, Climate Action, Systematic Literature Review (SLR)

## Abstract

**Background:**

This study examines the scientific misinformation about climate change, in particular obstructionist strategies. The study aims to understand their impact on public perception and climate policy and emphasises the need for a systemic understanding that includes the financial, economic and political roots.

**Methods:**

A systematic literature review (SLR) was conducted using the PRISMA 2020 model. The sample consisted of 75 articles published between 2019 and 2023, sourced from Web of Science, Scopus and Google Scholar. Methodological triangulation was performed to improve the analysis.

**Results:**

The results show that technological approaches to misinformation detection, such as immunisation and fact-checking, are widely used. However, few studies look in depth at the operational structures that support systematic disinformation.

**Conclusions:**

The study emphasises the urgent need to expand and deepen research on climate disinformation and argues for more global, comparative and adequately funded studies. It emphasises the importance of addressing the systemic complexity of disinformation and integrating different theoretical and methodological approaches. This will help to develop effective measures against hidden networks of influence and mitigate their disruptive effects. The research findings are relevant for policymakers, scientists, academics, the media and the public and will help to improve strategies to combat climate misinformation and promote science-based climate action.

## Plain language summary

This study examines how misinformation about climate change is disseminated and how it influences public understanding and policy decisions. The study focuses on the strategies used to create doubt and confusion about climate change. It emphasises the need to understand the financial, economic and political roots of these strategies.

We analysed 75 articles published in major academic databases between 2019 and 2023. Our aim was to find out how misinformation is recognised and countered. We found that most studies focus on technological solutions such as fact-checking and methods to build resistance to misinformation. However, there is a lack of research on the broader systems that support widespread misinformation.

Our study suggests that more comprehensive and globally coordinated research is needed. We call for studies that are better funded and cover more countries to effectively tackle the complex web of misinformation. This research is crucial for policy makers, scientists, journalists and the public. By understanding and addressing the causes of climate misinformation, we can develop better strategies to disseminate accurate information and support effective climate action.

## Introduction

The urgency of environmental issues ( Willis, 2020), including climate change and global warming ( IPCC, 2023), is part of a widespread concern and has been recognized as a key security challenge ( The National Intelligence Council, 2021a). This has led to numerous initiatives to mitigate its effects. The European Union is systematically promoting the transition from a linear economy to a circular, green and blue economy ( European Commission, 2022; European Commission, 2023), which requires public opinion to champion the associated change ( Carbonell-Alcocer *et al.*, 2022; Trudel, 2019).

Information and communication measures are crucial in this change process, as the success of this transition depends on a profound change in citizens' beliefs, attitudes, practices and behaviors ( Mcdonough & Braungart, 2002).

It therefore depends on cultural and cognitive processes that may encounter resistance to change, public inaction ( Hornsey & Fielding, 2020) and manipulative information tactics through various vectors that exploit the vulnerabilities and cognitive biases ( Soon & Goh, 2018) of societies, groups and individuals to spread narratives that question and contradict scientific knowledge, thus contributing to polarized reactions. Consequently, this can act as a powerful inhibitor of attitudes and behaviors that are susceptible to such change, limiting the effectiveness of science communication interventions that promote it and triggering a boomerang effect ( Palm *et al.*, 2017)

The spread of these narratives and the mechanisms and effects of scientific misinformation and disinformation have been widely studied in their most immediate processes and mechanisms, both in their cognitive effects and in the way they influence individual attitudes and behaviors.

However, more systematic approaches that assess from a critical perspective what are the main axes on which the ecosystem of scientific misinformation is built, and in particular the one that uses this as an obstruction strategy on climate change, are less common ( Almiron & Moreno, 2022). As studied ( Farrell *et al.*, 2019), macro-conditions that restrict or condition the creation and sharing of information can in themselves, without creating disinformation, favor its spread.

In the scientific field, we find both influence operations ( Larson *et al.*, 2009; Walton, 2019) and orchestrated campaigns to create confusion, spread misinformation or incomplete information based on the development of alternative narratives ( Roe & Shapira, 2021). These disinformation strategies and tactics have been used by business lobby groups such as the Competitive Enterprise Institute-funded Cooler Heads Coalition ( O'Harrow Jr., 2017) and industrialists, particularly the tobacco industry ( Glenza, 2019) and the fossil fuel industry ( Supran & Oreskes, 2017).

In the context of this study, systemic disinformation with financial, economic and/or political roots is considered to be the deliberate and coordinated dissemination of false or misleading information that originates in or is motivated by the strategic objectives and interests of relevant actors in these fields and influences public perceptions of climate change. It is considered systemic because it is part of a broader pattern or system designed or promoted by actors seeking to maintain, preserve or increase their financial benefits, influence the change and/or development of policies that benefit them economically, or achieve political or geostrategic objectives.

Systematic disinformation aimed at obstructing climate action, often based on the false rhetoric of climate activism ( Almiron & Moreno, 2022), has a profound impact on decision-making, public trust and the effectiveness of policies and the actions they promote. It is therefore important to know how scientific research approaches the study, what its findings are and what recommendations it makes for tackling climate change in its various dimensions.

### Background to scientific disinformation on climate change

The first mentions of disinformation and misinformation about climate change date back to the 1990s. The IPPC's Second Assessment Report on Climate Change, published in 1995, already contained in its foreword a general mention of the problem of misinformation as a tool that hinders the communication of environmental science. More specifically, we find accusations directly linking the hydrocarbon industry to the creation and promotion of climate disinformation campaigns. Several popular works are worth mentioning in this context ( McKibben, 1989; Gelbspan, 1997; Gore, 1993). At the end of the decade, authors such as Ehrlich *et al.*, (1999) stated that disinformation was the main obstacle to the creation of knowledge about climate, despite the enormous amount of information that has been generated about environmental problems in recent decades.

On the other hand, when reviewing the scientific literature, we can see that time and again an extensive and undifferentiated use of concepts is made, leading to greater confusion about the nature of the phenomenon at a systemic level. This can be observed with the terms disinformation and misinformation, which are often used indiscriminately without referring to or attributing the malicious nature of disinformation.

Similarly, the concept of denial has been used as a catch-all term to summarize all attitudes that question the scientific consensus on climate change. Far from this ascribed uniformity, authors such as Almiron & Moreno (2022) warn of the need to create different categories that represent the diversity of attitudes held in order to understand the complexity of the phenomenon: skepticism (doubts about climate science and the attribution of pseudoscientific positions), countermovement (uncoordinated actions aimed at undermining climate science and policy), contrarianism (opposition to climate action), retardism (the term applied to those who sow doubt about action to mitigate climate change and call for inaction), and obstructionism (actions aimed at hindering policies to reduce pollution and emissions). Of all of them and given the increasing recognition of the existing scientific consensus on climate change and the direct experience of the effects of global warming ( Leiserowitz *et al.*, 2023), obstructionism is gradually taking shape as the dominant approach with a distinctly ideological character, whose common factor is the defense of capitalism rather than the denial of climate change itself ( Almiron & Moreno, 2022).

### Obstructionism on climate action as a strategy

There is no doubt that misinformation and misinformation about climate change is currently a recognized threat because it can prevent necessary decisions ( United Nations, 2023).

Much of the scientific literature on the subject addresses the problem of skepticism ( Haltinner & Sarathchandra, 2023) and climate change denial and, from a more complex and choral perspective of the problem, the obstruction of climate action and the strategies developed by the promoting actors to sow confusion in public opinion about the existence of climate change, the deconstruction of the arguments used, their scope and their impact on the public. As Almiron and Moreno point out, "obstructionism is both a narrative and a strategy. In both cases, the goal is the same: to delay policies aimed at reducing greenhouse gas emissions or pollution in general because they are seen as a threat to the economy." ( 2022, p. 14) From a broad perspective, obstructionism is described as a process of ideological mediation ( Martin Serrano, 1977; Ricoeur, 1994) in which those social representations that favour inaction are constructed by using mental frames ( Lakoff, 2008) and selecting those social representations that can evoke them ( Berger & Luckmann, 1966). Other recent studies have assessed the role of specialised journalism in combating climate misinformation by recognising verification patterns and identifying common narratives and actors in the dissemination of false content ( Fernández-Castrillo & Magallón-Rosa, 2023), and others provide a detailed analysis of the way obstructionist arguments are amplified via platforms such as YouTube, illustrating the complex interplay between denial and obstructionism in climate change discourse ( Olmeda, 2022)

The basis of obstructionism is the financial, economic and political roots that influence polarization on environmental issues to promote negative, delaying or adversarial discourses, among others, through organizations funded by political and financial actors with strategic interests. In its paradigmatic dimension, its function is to construct a shared social imaginary ( Castoriadis, 1975) as an alternative to scientific knowledge, using the interrelation between this and the individual mental system to create a shared sense and feeling that binds social groups and collectives together and can influence public opinion. These influence operations were investigated by Farrell (2016) using computational methods. This uncovered the institutional structures that fund and coordinate scientific disinformation campaigns ( Goldman *et al.*, 2017) and include "think tanks, philanthropic foundations, corporations, trade associations, advocacy groups, front organizations, front companies, lobbyists, and public relations firms" ( Dunlap & McCright, 2011).

Numerous cases are documented in the literature. González & Agudo (2000), based on an earlier analysis by ( Ong & Glantz, 2000) of the tobacco cartel's decades-long disinformation operations, identified the keys to manipulation and corruption in the scientific field, which they divided into five areas: (a) hiring scientific consultants to obtain information about the studies conducted in order to expose possible biases or weaknesses in their design and conduct and jeopardize their integrity; (b) encouraging the development of review articles and publications arising from scientific meetings that would lead to contrary conclusions or create confusion; (c) Funding collaborative agreements to influence studies; (d) Challenging the role of the discipline, in this case epidemiology, as a basis for public health policy; (e) Promoting alternative frameworks, e.g. through the development of a code of ethics for health professionals. E) promoting alternative frameworks, e.g. through the development of a code of good epidemiological practice, that would limit the use of science in decision-making.

These practices have also been observed in the oil industry for decades. In the United States, the House Oversight Committee has documented that oil companies have known about the impact of their operations on climate change and global warming since the 1980s ( Gramling, 2021) and how they have used disinformation as a strategy to evade their responsibilities and further increase their profits ( Powell, 2021). Supran (2021) has identified three keys in ExxonMobil's rhetoric to disinform and create an alternative narrative, highlighting the similarity to the tobacco model: 1) downplaying the seriousness of climate change; 2) demonstrating the value, importance and necessity of fossil fuels for prosperity and progress; 3) blaming consumers for climate change.

As the fourth edition of the Production Gap Report ( SEI, Climate Analytics, E3G *et al.*, 2023) points out, the environmental rhetoric and greenwashing of these companies is not matched by effective action. In fact, governments' commitment to reducing fossil fuel production is low. They plan to produce twice as much as would be needed to limit warming to 1.5°C.

### Disinformation as a counterargument

Paradoxically, we observe the use of the term disinformation as an argumentative strategy to disqualify climate science. The positioning of these counterarguments in the public sphere is part of the struggle to shift the focus of the Overton Window in relation to climate change, as in the face of public positions in favor of tackling the problem ( Turrentine, 2019), others contribute to distrust in scientific knowledge, the rise of denialism, and entrenchment in authoritarian attitudes ( Herranen, 2023; Reay, 2016).

As Haltinner & Sarathchandra (2023) have pointed out, climate change skepticism is a complex social phenomenon, not just a scientific one, which has an extraordinary diversity and therefore needs to be understood to be acted upon.

We find its beginnings in 1977 in the statements of the president of an aerosol company defending himself against criticism of hydrofluorocarbons, denouncing their origin in a "campaign "orchestrated by the KGB's Ministry of Disinformation" ( Gordon & Suzuki, 1991, p. 25). Later, in the early 1990s, the Competitive Enterprise Institute noted in a report that the Environmental Protection Agency (EPA) was "promoting irrational fears for its own benefit through disinformation" ( Smith, 1992, p. 4).

At other times, the concept is used as a weapon to discredit key proponents of climate change. This strategy is evident in literature reviews such as the review of "Earth in the Balance: Ecology and the Human Spirit" by Albert Gore Jr. (1993). In this review, the vice president is portrayed in a kind of simplistic and Manichean characterization in which the denialist lobby is portrayed as "self-interested cynics . . . seeking to cloud the underlying issue of the environment with disinformation," in contrast to the climate change lobbyists who are portrayed are romantically" ... "They appear only as individual men and women of humble backgrounds, members of the environmental "resistance" ( Hahn, 1993, p. 1744).

Similarly, Gelbspan's acclaimed 1997 work, "The Heat Is On: The High Stakes Battle over Earth's Threatened Climate, has been criticized for the author's allegedly unfounded accusations of disinformation against the hydrocarbon industry because "the organization and funding of the campaign are not detailed and it is all too easy to defame those who question the global warming arguments as tools of big business" ( Stolley, 1999, p. 235).

Another strategy of climate change deniers or sceptics is to accuse the representatives of the so-called alarmist lobby of an unjustifiably fatalistic discourse and an exaggeration of the extent and effects of climate change. This strategy can be found in some representatives of mass literature and in the work of conservative think tanks. Particularly noteworthy in this context is the work Red Hot Lies: How Global Warming Alarmists Use Threats, Fraud, and Deception to Keep You Misinformed by Christopher Horner (2008), which also outlines the neoconservative background to these positions, which opposes environmental regulations that are justified by deliberately magnifying the scale of the climate crisis in order to promote growth and economic prosperity.

This same neoconservative line seems inescapably linked to the political propaganda of Donald Trump's presidential campaign and was crucial to his 2016 election victory ( Abudu *et al.*, 2023). During this period, the conservative candidate forged a populist narrative around climate change mitigation measures, portraying them as an obstacle to the development of capitalism and a competitive disadvantage for the US in favour of countries such as China and India, which would have been guaranteed deadlines for the energy transition ( Nordensvard & Ketola, 2022). In campaign speeches, we often find qualifiers such as "anti-worker" and "job-destroying" ( Abudu *et al.*, 2023) for these regulations because they would harm the working class because of the implied global redistribution efforts. In this way, Trump's political propaganda pushed the public against candidates who care about climate change ( Bovet & Makse, 2019).

But also in the United States, the Intelligence Community has assessed in its assessment "Climate Change and International Responses Increasing Challenges to US National Security Through 2040" that "climate change will increasingly exacerbate risks to US national security interests as physical impacts increase and geopolitical tensions rise over how to respond to the challenge" ( National Intelligence Council 2021b: i).

### Agents promoting and disseminating denialism

These arguments were debunked when it became known that the hydrocarbon industry had been funding the denialist lobby for decades. This evidence came to light through lawsuits against companies, information obtained through Freedom of Information Act (FOIA) requests ( Mulvey *et al.*, 2015), and through disclosure by researchers who had access to the originals, as well as by the Royal Society and the Union of Concerned Scientists (UCS). Analysis of the documents ( Gelbspan, 1997; Oreskes, 2011; Oreskes & Conway, 2011) showed that the major industrial companies (Chevron, ConocoPhillips, ExxonMobil, BP, Shell and Peabody Energy) had been directly and persistently involved in disinformation campaigns aimed at distorting reality about the risks of climate change through trade associations and front organizations for decades. Later, The Guardian documented in a detailed report how the fossil fuel industry had deliberately misled the American public about climate change ( Cook *et al.*, 2019).

Fundamental to the success of this strategy was the role of conservative think tanks funded by industry through affiliated foundations. According to the analysis of denialist literature conducted by Jacques *et al.*, (2008), of the 141 books published between 1972 and 2005 that promoted environmental scepticism, 130 were associated with conservative think tanks. Their geographic origins were widely dispersed and included Australia, Canada, Europe, and South Africa in addition to the United States ( Jacques *et al.*, 2008). Their large-scale organizational capacity was key to countering the initial success of the environmental movement in the 1970s and 1980s. The list of these think tanks includes Competitive Enterprise Institute, Heratland Institute, Marshall Institute, Cato Institute, American Enterprise Institute, Claremont Institute, National Center for Policy Analysis, National Center for Public Policy Research, Centers for a Sound Economy Foundation, Reason Public Policy Institute for Research on the Economy and the Environment, or the Pacific Research Institute ( McCright & Dunlap, 2010).

At the same time, the traditional media and, more recently, the new digital channels act as vectors for the spread of disinformation. The work of Supran & Oreskes (2020) is revealing in this respect. It shows how ExxonMobil paid the New York Times to publish editorial-style advertisements between 1989 and 2004. The strategy of sowing doubt about climate science was in turn facilitated by the norm of balanced media coverage, where the different sides of the climate debate were equally represented ( Dunlap & McCright, 2010).

The rise of social media in recent years has in turn contributed to the exponential spread of climate change disinformation, acting as an amplifier of denialist and obstructionist discourse and reaching an even wider audience. Creating confusion about the scientific consensus on the existence of climate change has become the fundamental goal of the denialist movement, mainly thanks to the use of automated bots that have reinforced the existing polarization ( Chen *et al.*, 2021). In this sense, Marlow, Miller and Roberts (2021) have systematized the main mechanisms through which social networks contribute to the polarization of discourse: a) exposure to uncivil conversations about controversial content, leading to increased affective polarization; b) configuration of a fragmented information environment, which reduces the overall quality and favours the inclusion of marginal viewpoints; and c) exposure of users to a greater number of opposing viewpoints, which can lead to the activation of boomerang-like responses or the hostile environment.

### Strategies for combating scientific misinformation on climate action

Some of the literature has dealt with possible solutions to counteract these malicious or false information flows. A variety of aspects, knowledge of which is necessary for a comprehensive understanding of the phenomenon, and which requires multidisciplinary approaches from different perspectives and academic disciplines: from psychology, computer science, political science, pedagogy and communication ( Cook *et al.*, 2015).

Farrell *et al.*, (2019) propose four main strategies to combat scientific disinformation in this area based on an analysis of how disinformation campaigns are developed as part of a multi-layered strategy to create a thematic agenda and ideological focus on public opinion.

The first of these measures is public vaccination. The reception of scientific information is shaped by pre-existing ideologies and beliefs, so it is formed through a process of "cultural cognition". Therefore, it is not enough to point out the facts or refer to the scientific consensus, because in the "post-truth" era the value of evidence is weakened in favor of established beliefs, and it is easy to stir up skepticism in this context. An alternative approach is "attitudinal inoculation" with inoculation models against disinformation that anticipate the reaction to it. One way to do this is to provide data on who is behind and funding the media that spread disinformation.

The second is to develop legal strategies. In the United States, multi-million-dollar lawsuits against industries, particularly oil companies such as ExxonMobil, have exposed how the company concealed data from its own reports on the impact of its operations on climate change for years ( Supran & Oreskes, 2017). Multi-million-dollar judgments, as in the case of the tobacco industry or the chemical industry such as Dupont ( Bilott, 2020), despite their complexity, have an important corrective effect on the way this issue is handled by large companies.

The third possibility is the use of political channels and mechanisms. The politics of polarization has found its niche in social discontent by exploiting, especially through social networks, politically based scientific disinformation arguments that better fit certain beliefs: such as energy independence, deregulation or nationalism and non-interference, as well as the economic interests of certain collectives that see their lucrative businesses threatened. These strategies are covert communication measures that finance seemingly neutral civil movements. Or they use the social structures of religious organizations to spread or stop certain initiatives.

Finally, financial transparency must be promoted. It is necessary to make funding from private philanthropists and industry transparent so that we know who they are funding and for what purposes. As mentioned earlier, large corporations are behind the groups spreading scientific disinformation about climate change. So the opacity enabled by mechanisms such as foundation funding, which protect the identity of donors, must be avoided.

Misinformation has become the dominant strategy of the climate denier, feeding a limited understanding of climate change in the scientific literature and in public opinion. Since the 1990s, disinformation and misinformation promoted by the hydrocarbon industry has been studied as a method to obstruct scientific communication about climate change, its causes and consequences. Despite the vast amount of information available, the published scientific literature has not helped to reduce the confusion, likely due to the limited scope and focus of the studies conducted.

### Objectives

Disinformation processes in the context of climate change represent a complex and multidimensional problem that, while having local characteristics, also has systemic and global features that contribute to hindering climate action.

The overall aim of this review is to understand, based on a systematic review of the scientific literature (SLR), how research on this subject of investigation is approached considering the climate action obstructionism approach and what are the characteristics of the research conducted to assess whether its results provide actionable information that enables decision making aimed at improving interventions to promote sustainability and the circular economy.

The specific objectives and research questions are listed in Table 1.

**Table 1.** Relationship between objectives and research questions.

Specific objectives	Research questions
SQ1. Check whether the objects of study and approaches of climate disinformation research are approached systemically.	R.Q.1.1. What are the **objects of study**?
	R.Q.1.2. What are the **theories** that make up the theoretical framework from which these studies are approached?
SQ2. Characterize the research in terms of the predominant themes and methods.	R.Q.2.1. What **methodologies** are applied in the analysis of the information?
	R.Q.2.2. What are the **data collection** and **analysis techniques**?
SQ3. Evaluate the results and purpose of the studies referred to.	R.Q.3.1. What is the nature of the **conclusions**?
	R.Q. 3.2. Are **solutions** and/or **operational** **recommendations** given in the studies addressed?

**Figure 1.**
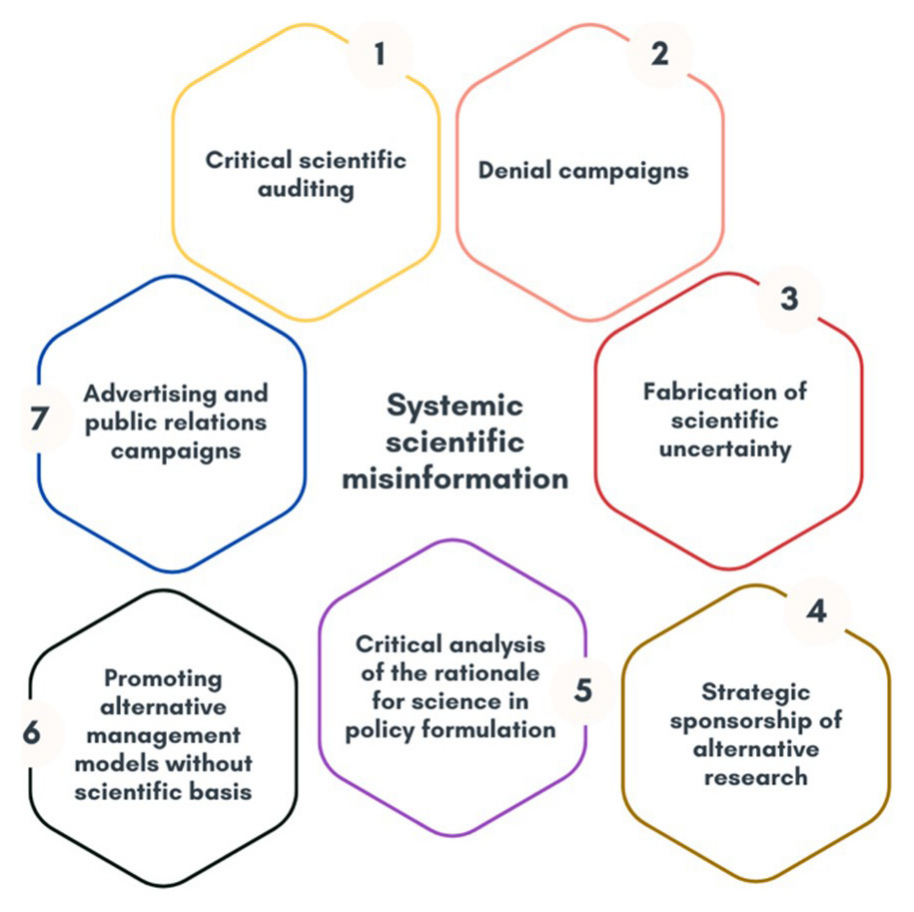
Systemic scientific misinformation. Own elaboration based on data from González and Agudo (2000).

As a general framework for analysis, a matrix of systemic disinformation with financial, economic, and political roots is drawn up, the details of which are shown in Figure 1 and Table 2. It was developed from the analysis of global approaches that mainly reflect the literature reviewed in the state of the art, which includes scientific articles as well as technical reports, dissertations and working papers.

**Table 2.** Definition of concepts.

Concept	Description
Critical scientific auditing	Detailed analysis of solid scientific studies by experts hired for the express purpose of discrediting climate scientists. The analysis focuses on minor weaknesses or deliberately misleading or biased interpretations of data to cast doubt on the validity of the research.
Denial campaigns	Strategy for the development of communication measures to question or deny the existence or seriousness of anthropogenic climate change.
Fabrication of scientific uncertainty	Strategy to promote the production and dissemination of scientific literature with the explicit aim of sowing doubt and confusion about scientific topics on which there is a clear, established consensus, to generate alternative narratives despite the existence of indisputable scientific evidence. This includes the promotion of alternative narratives by pseudo-experts and false experts in climate science who spread conspiracy theories or views that contradict established scientific evidence.
Strategic sponsorship of alternative research	The process by which entities or individuals with vested interests provide funding to conduct research to direct or influence the results of that research in a way that supports their own goals or agendas. This includes cherry-picking, which emphasizes data or studies that support hidden agendas while ignoring or minimizing scientifically sound data and results.
Critical analysis of the rationale for science in policy formulation	Sceptical evaluation of the knowledge, data, theories and results of climate science to influence public policy on climate change. This includes attacking the scientific consensus and integrity of climate scientists by introducing information that may cast doubt on their motives.
Promoting alternative management models by restricting the weight of science in policy formulation and decision making	Develop and promote alternative approaches to environmental management that prioritize practices that are considered best or most effective from a practical or ethical perspective, and that reduce or mitigate the role of scientific evidence in policy and administrative decisions. This approach may include the adoption of codes of conduct, guidelines or standards that emphasize non-scientific criteria such as community values, traditional knowledge or economic considerations in the planning and implementation of environmental measures. It also includes the deliberate obstruction of policy and legislation.
Advertising and public relations campaigns	Develop PR campaigns to improve the image of actors that hinder climate action to divert attention from their impact on climate change. This includes actions that show that they are committed to sustainable solutions even though they continue to use harmful practices.

### Hypothesis

The research proposes the following hypotheses:

**H1.** Studies that systematically and comprehensively address climate change disinformation, incorporating analysis of its financial, economic, and political roots, provide a deeper and more effective understanding of the phenomenon and enable the development of more effective interventions to counter the obstruction of climate action (R.Q.1.1 and R.Q.1.2).

**H2.** Research that utilizes diversified data collection and analysis methods and techniques tailored to the complexity of the climate disinformation phenomenon is better able to identify and propose effective operational solutions and recommendations to combat disinformation and promote sustainability and circular economy (R.Q.2.1 and R.Q.2.2)

**H3.** Studies that can critically evaluate results and offer solutions or operational recommendations based on a systemic and comprehensive understanding of financial, economic and political climate disinformation contribute significantly to the formulation of more effective policies and measures to address climate action obstruction (R.Q.3.1 and R.Q.3.2).

## Methods

**Figure 2.**
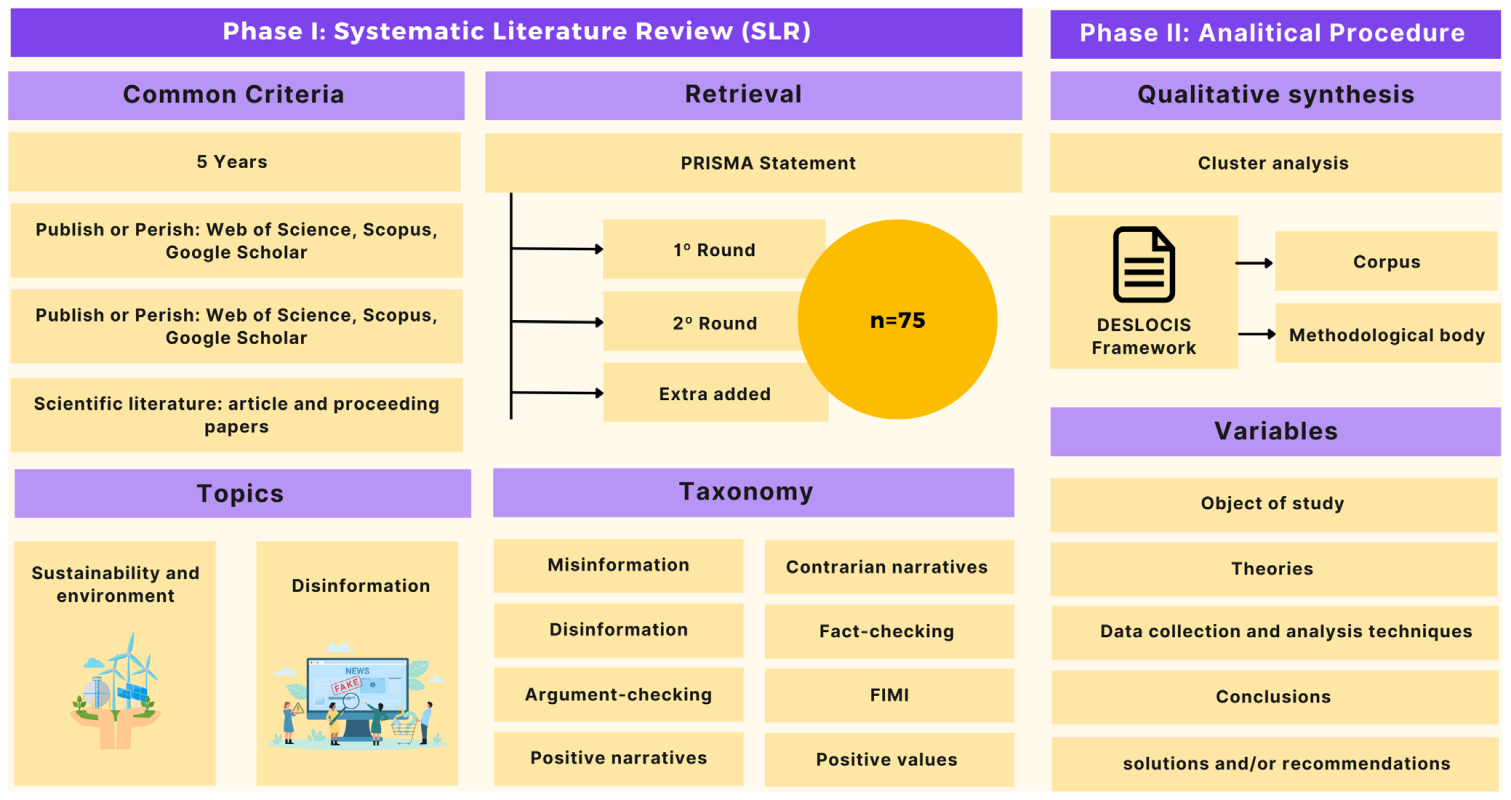
Methodological approach of the research.

The methodology used was a systematic literature review (SLR) using the Preferred Reporting Items for Systematic Reviews and Meta-Analysis (PRISMA). Specifically, we used the PRISMA 2020 flow diagram and checklist ( Page *et al.*, 2021). Multiple researchers are involved in the process of selecting and reviewing publications to reduce bias ( Denzin, 2017). The process is divided into two phases. The first focuses on finding and classifying the scientific literature and the second on the qualitative and quantitative analysis of the selected documents. Figure 2 summarizes the methodological approach of the research in general terms:

### Phase I: Systematic Literature Review (SLR)

**Figure 3.**
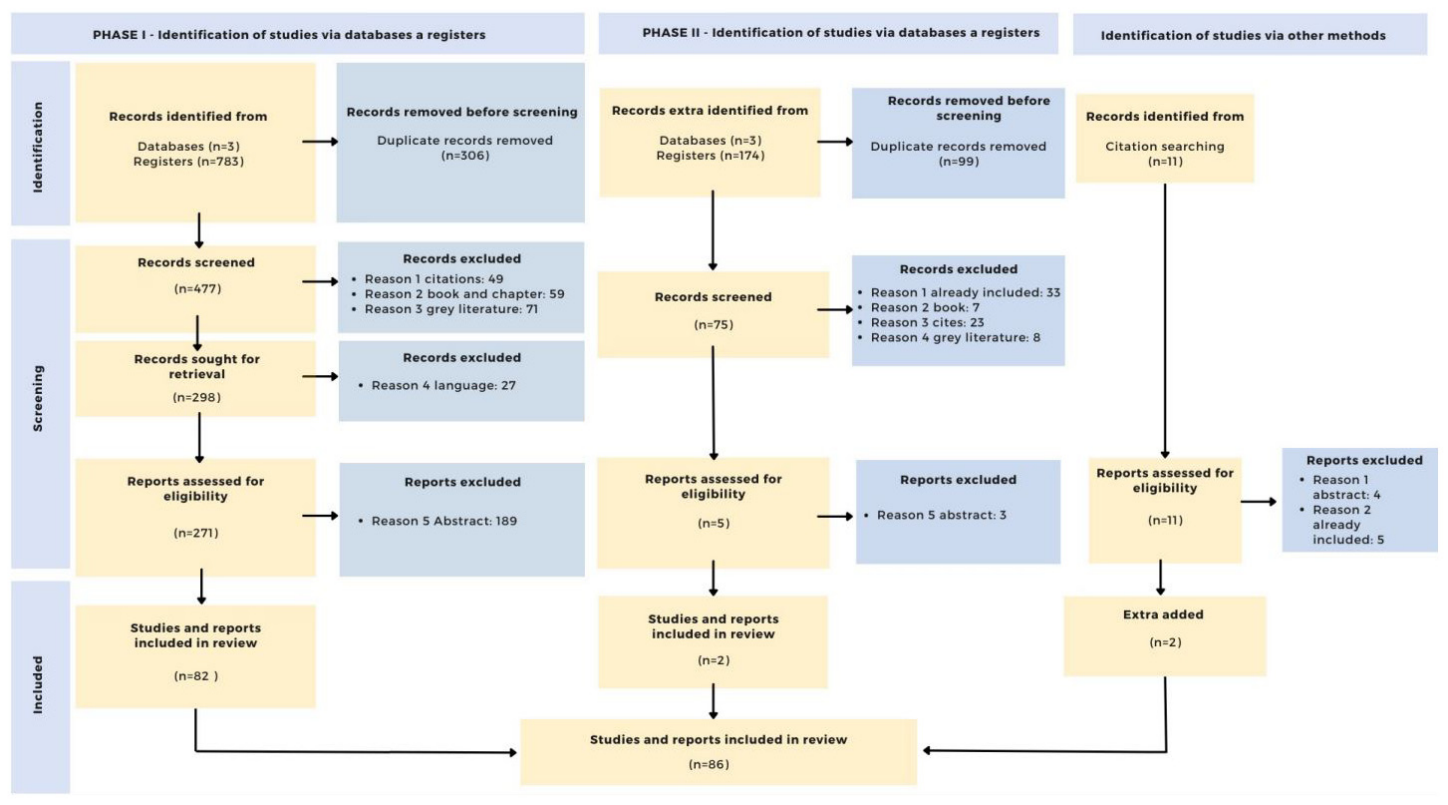
PRISMA Flow diagram.

The search protocol is carried out twice, with the terms being adjusted to avoid documentary noise and to answer the research questions more precisely. The general criteria for the literature search and the two search rounds are described in detail in the following sections. Figure 3 shows the PRISMA procedure in detail.

### Review approach: general criteria

First, the scope of the research is limited to the academic literature of the last five years dealing with sustainability issues in the field of misinformation, according to the research questions and objectives set out in Section 3.

The aim is to know the relevance of the studies conducted and examine their trend approach. The research aims to analyse the scope of the object of study: whether it is a reduced corpus, and the publications tend to a micro approach of a specific case or are derived. In contrast, studies could be based on research projects with a broad scope of the problem, with a sizable corpus and/or several related publications. Large-scale studies may be interesting in terms of knowledge of the problem. Based on documentary sources, they would provide an overview of the current situation. However, one must first find out what has been researched and how.

One aspect to consider is related to the interventionist motivation of the research conducted, so that the observation of proposals or possible solutions provided by the research is also captured in the review. For empirical studies based on secondary sources, the question arises as to how they can be scaled and whether their results can be operationalized.

The lack of studies that focus on identifying the formal characteristics of research and evaluating them in a generalized way presents an opportunity to analyse scientific publications ( Carbonell-Alcocer *et al.*, 2021). The steps of the systematic literature review include determining the scope and research questions, defining the selection criteria, critically appraising the selected papers using a systematic pathway, synthesizing and qualitatively and/or quantitatively analysing the content of the articles, and finally presenting the assessments in a system selected to adequately answer the designed study questions ( Hafezi *et al.*, 2018). These aspects are examined to determine an appropriate search term in each database, as actual or potential overlaps may have been overlooked. The proposed systematic review provides an updated inventory of the research phenomenon surrounding the object of study ( Homar & Cvelbar, 2021).

The search procedure will be carried out using the Publish or Perish software version ( Harzing, 2007) from the institutional network of the Universidad Rey Juan Carlos. This software makes it possible to identify the literature from the different databases and search engines according to predetermined criteria. To standardize the search, the title was defined as a common field for the databases in which the search was to be carried out. The criteria set were to find the scientific literature of the last 5 years from Web of Science, Scopus and Google Scholar (2019 to mid-2023).

The search method is based on Boolean operators that combine terms from the environment and sustainability domain with terms from the misinformation domain. The terms from the first area are selected based on the descriptors of the eComciencia project (2023). The terms from the area of misinformation were selected by experts in the field who have extensive experience with research projects in this area.

### Recovery process

The search for scientific literature with PRISMA is explained below. This was done in two steps to make the collection of information as precise as possible. In addition, documents that had been identified in other ways were also added due to the specific nature of the subject area.

***First PRISMA: identification***

First, 6 general terms from the area of environment and sustainability and 11 specific terms from the area of disinformation were selected. Table 3 summarizes the selected keywords for the first PRISMA search, and Table 4 shows the Boolean operators used in the first search. By combining the words, 12 Boolean operators are generated.

**Table 3.** Search areas first PRISMA.

Ámbito 1: environment and sustainability		
“Sustainable development”	“Environmental education”	“Environmental awareness”
“Education for sustainable development”	“Bioenergy”*	“Climate change”
Ámbito 2: disinformation		
“Information manipulation”	“Fake news”	“Foreing influence”
“Informative influence”	“Fact checking*”	“Debunking*”
“Disinformation”	“Narratives”	“Inoculation”
“Misinformation”	“Countermeasures”	-

**Table 4.** Boolean operators first search. By combining the words, 12 Boolean operators are generated.

ID	Boolean operators
**B1**	“Sustainable development” AND (“Information manipulation” OR “Informative influence” OR “Disinformation” OR “Misinformation” OR “Fake news”)
**B2**	“Education for sustainable development” AND (“Information manipulation” OR “Informative influence” OR “Disinformation” OR “Misinformation” OR “Fake news”)
**B3**	“Environmental education” AND (“Information manipulation” OR “Informative influence” OR “Disinformation” OR “Misinformation” OR “Fake news”)
**B4**	“Bioenergy”* AND (“Information manipulation” OR “Informative influence” OR “Disinformation” OR “Misinformation” OR “Fake news”)
**B5**	“Environmental awareness” AND (“Information manipulation” OR “Informative influence” OR “Disinformation” OR “Misinformation” OR “Fake news”)
**B6**	“Climate change” AND (“Information manipulation” OR “Informative influence” OR “Disinformation” OR “Misinformation” OR “Fake news”)
**B7**	“Sustainable development” AND (“Fact checking*” OR “Narratives” OR “Countermeasures” OR “Foreing influence” OR “Debunking*” OR “Inoculation”)
**B8**	“Education for sustainable development” AND (“Fact checking*” OR “Narratives” OR “Countermeasures” OR “Foreing influence” OR “Debunking*” OR “Inoculation”)
**B9**	“Environmental education” AND (“Fact checking*” OR “Narratives” OR “Countermeasures” OR “Foreing influence” OR “Debunking*” OR “Inoculation”)
**B10**	“Bioenergy”* AND (“Fact checking*” OR “Narratives” OR “Countermeasures” OR “Foreing influence” OR “Debunking*” OR “Inoculation”)
**B11**	“Environmental awareness” AND (“Fact checking*” OR “Narratives” OR “Countermeasures” OR “Foreing influence” OR “Debunking*” OR “Inoculation”)
**B12**	“Climate change” AND (“Fact checking*” OR “Narratives” OR “Countermeasures” OR “Foreing influence” OR “Debunking*” OR “Inoculation”)

This first search was carried out in May 2023 (May 19) via the institutional network of the Universidad Rey Juan Carlos. In this first phase, a total of 783 records were retrieved and found in the first Zenodo archive ( Gertrudix *et al.*, 2024a). The Table 5 summarizes the relationship between Boolean, databases and search engines and the number of records retrieved.

**Table 5.** Records retrieved first search.

	B1	B2	B3	B4	B5	B6	B7	B8	B9	B10	B11	B12	Total
**Scopus**	0	0	1	0	0	34	44	0	10	3	2	134	228
**Wos**	0	0	0	0	0	23	17	0	11	3	0	82	136
**Scholar**	3	0	1	0	1	97	100	2	14	0	1	200	419
**Total**	3	0	2	0	1	154	161	2	35	6	3	416	**783**

***First Prisma: screening and included***

Next, repeated documents are eliminated, leaving 477 records. Next, citations (n=49), book chapters (n=59) and gray literature (bachelor's and master's theses) (n=71) are eliminated.

With the full list of records (n=298), the documents are retrieved and those that are not in English (n=27) are eliminated. In addition, the data is curated by retaining the title, authors, year of publication, source, doi and citations in Web of Science, Scopus and Google Scholar. After this process, 271 documents are retained. The data corresponding to this process can be found in the second Zenodo archive ( Gertrudix *et al.*, 2024a).

To check whether the documents really refer to the topics studied, the summaries of the 271 documents retrieved from different researchers are reviewed. To facilitate the analysis of the results, the documents are categorized into two levels at this point: Major Category and Minor Category. The terms used to perform the classification are:

1. Disinformation
2. Foreign Information Manipulation and Interference
3. Misinformation
4. Fact-checking
5. Argument-checking
6. Contrarian narratives
7. Positive narratives
8. Positive values

After this process, 189 documents were excluded and 82 documents were retained. The data for this phase can be found in the third Zenodo file ( Gertrudix *et al.*, 2024a). In addition, the data on the selected publications are expanded in the matrix to include the authors' names, position in the company, institution, area of knowledge and gender.

When reviewing the abstracts, it was found that the terms countermeasures, vaccination and narratives generate a lot of documentary noise. This leads to documents being included in the original matrix that have nothing to do with the topic. For this reason, it was decided to conduct a second search to refine the results.

***Second PRISMA: Identification***

By combining the words, 6 Boolean operators are formed:

A total of 174 documents were retrieved from Google Scholar (75), Web of Science (20) and Scopus (79).

This second search will be carried out in June 2023 (June 8) through the institutional network of the Universidad Rey Juan Carlos.

In this second phase, a total of 174 documents were retrieved. The data corresponding to this phase can be found in the fourth Zenodo archive ( Gertrudix *et al.*, 2024a). Table 6 summarizes the search areas for the second PRISMA, including the terms used. Table 7 shows the Boolean operators used in the second search, and Table 8 details the number of records retrieved from each database and search engine.

**Table 6.** Search areas second PRISMA. In this second phase, the following terms are used.

Area 1: environment and sustainability		
*Climate change*	*Sustainable development*	*Climate justice*
*Ecojustice*	*Climate migration*	*Climate trauma*
Area 2: misinformation		
*Disinformation*	*Misinformation*	*Fake news*
*Dominant Narratives*	*Information manipulation*	*Possitive argument*
*Believes*	*Hostile narratives*	*Non-state actors*

**Table 7.** Boolean operators second search.

ID	Boolean
**SBE1**	"Climate change" AND ("Disinformation" OR "Misinformation" OR "Fake new" OR "Dominant Narrative" OR "Information manipulation" OR "Positive argument" OR "Believes" OR "Hostile narrative" OR "Non-state actor")
**SBE2**	"Sustainable development" AND ("Disinformation" OR "Misinformation" OR "Fake new" OR "Dominant Narrative" OR "Information manipulation" OR "Positive argument" OR "Believes" OR "Hostile narrative" OR "Non-state actor")
**SBE3**	"Climate justice" AND ("Disinformation" OR "Misinformation" OR "Fake new" OR "Dominant Narrative" OR "Information manipulation" OR "Positive argument" OR "Believes" OR "Hostile narrative" OR "Non-state actor")
**SBE4**	"Ecojustice" AND ("Disinformation" OR "Misinformation" OR "Fake new" OR "Dominant Narrative" OR "Information manipulation" OR "Positive argument" OR "Believes" OR "Hostile narrative" OR "Non-state actor")
**SBE5**	"Climatic migration" AND ("Disinformation" OR "Misinformation" OR "Fake new" OR "Dominant Narrative" OR "Information manipulation" OR "Positive argument" OR "Believes" OR "Hostile narrative" OR "Non-state actor")
**SBE6**	"Climate trauma" AND ("Misinformation" OR "Fake new" OR "Dominant Narrative" OR "Information manipulation" OR "Positive argument" OR "Believes" OR "Hostile narrative" OR "Non-state actor")

**Table 8.** Records retrieved second search.

	SBE1	SBE2	SBE3	SBE4	SBE5	SBE6	TOTAL
**Scopus**	75	2	2	0	0	0	79
**WoS**	20	0	0	0	0	0	20
**Scholar**	74	1	0	0	0	0	75
**Total**	169	3	2	0	0	0	**174**

***Second PRISMA: screening and included***

After eliminating duplicate documents (n=99), the same criteria as in the first search were applied to the remaining 75. Thus, citations (n=23), book chapters (n=7), gray literature (bachelor's and master's theses) (n=8) and documents that were included in the first search (n=33) were eliminated. This process can be found in the fifth Zenodo archive ( Gertrudix *et al.*, 2024a).

Next, the abstracts (n=5) are reviewed. 2 documents are retained, and 3 documents are excluded. The data for this last phase can be found in the sixth Zenodo archive ( Gertrudix *et al.*, 2024a).

Identification of studies via other methods

During the review of the documents, further studies were identified (n=11) that could be related to the topic under investigation. These were analyzed and 2 documents were retained, excluding those that were already included (n=5) and those that were not related to the research questions (n=4). The data for this phase can be found in the seventh Zenodo file ( Gertrudix *et al.*, 2024a).

After completion of the identification and screening phase, the documents from the first (n=82) and second (n=2) searches as well as the documents related to other routes (n=2) are added. Thus, 86 documents are part of the final review. These can be found in the eighth Zenodo archive ( Gertrudix *et al.*, 2024a).

The selected articles, together with their bibliometric data, are included in a single data matrix and the full documents are downloaded. After evaluation, documents that are not accessible or do not fulfill any of the above criteria are excluded (n=11). Thus, 75 documents are included in the analytical synthesis.

The following diagram summarizes the entire process of selection, refinement and inclusion of publications ( Gertrudix *et al.*, 2024b).

### Phase II: Analytical procedure

The analysis procedure is divided into two phases, a qualitative and a quantitative phase.

The qualitative phase aims to analyse the corpus of the selected publications (n=75) to determine the formal and methodological characteristics of the studies. Table 9 shows the relationship between study categories and applied methodologies. For this purpose, the DESLOCIS ("Descriptors for a systematic literature review on social sciences") analysis framework ( Gertrudix, *et al.*, 2021) will be used in an online form completed by the researchers themselves. This model has already been used in other research in sustainability and circular economy to identify the maturity of the scientific literature ( Carbonell-Alcocer *et al.*, 2023; Romero-Luis *et al.*, 2021).

**Table 9.** Relationship between study categories and applied methodologies.

Category	Predominant type of research	Focus	Type of sample	Sample Size	Examples
Processes of disinformation and misinformation on climate change	Analytical and comparative	Mainly focused on disinformation and misinformation	Intentional, probabilistic and structural sampling	From less than 50 to more than 2000	"The Relationship between Bullshit Receptivity and Willingness to Share “Misinformation about Climate Change" and "Twitter's Fake News Discourses Around Climate Change and Global Warming".
Analysis of positive and counter narratives on climate change.	Descriptive and analytical	They explore positive and counter narratives, focusing on how they are constructed and perceived.	Intentional or convenience sampling	Generally small sample sizes	"Narratives of Hope: Imagination and Alternative Futures in Climate Change Literature" and "Climate change narratives in Philippine print news media".
Fact-checking y argument-checking sobre informaciones relacionadas con el cambio climático	Mostly analytical and comparative	Focused on verifying facts and arguments related to climate change.	Intentional and probabilistic sampling	Samples ranging from small to large	"Counteracting French Fake News on Climate Change Using Language Models" and "Fact-checking Climate Change: An Analysis of Claims and Verification Practices by Fact-checkers in Four Countries".
Educational and communicative strategies to fight climate skepticism and denialism	Descriptive and analytical	Focused on how communication and education can influence the perception of climate change.	Intentional sampling	Generally medium to large samples	"Education in the Anthropocene: Disinformation and power relations on environmental education" and" Using stories, narratives, and storytelling in energy and climate change research".
Educational and communicative strategies to fight climate skepticism and denialism	Analytical and explanatory	They examine how inoculation techniques can combat misinformation.	Intentional or significant sampling	Medium to large samples	"Inoculation, Climate Change and aPost-Factual, Hyperreal World?" and "Active versus passive: evaluating the effectiveness of inoculation techniques in relation to misinformation about climate change".
Models of climate change disinformation propagation through social networks and technological platforms.	Analytical and descriptive	They focus on the dissemination of disinformation through social networks and technological platforms.	Purposive or structural sampling	Different sample sizes.	"Effects of fact‐checking warning labels and social endorsement cues on climate change fake news credibility" and "Characterizing the semantic features of climate change misinformation on Chinese social media".
Individual narratives and perceptions on climate change	Analytical and descriptive	They explore how personal narratives and individual perceptions affect understanding of climate change.	Generally with intentional sampling.	Small to medium samples	"Urban climate change, livelihood vulnerability and narratives of generational responsibility in Jinja, Uganda" and "Narrative Empathy: A Narrative Policy Framework Study of Working-Class Climate Change Narratives and Narrators".

In the quantitative phase, an analytical process is carried out using a cluster analysis of the variables contained in the research questions. The online form and the results from the quantitative phase are included in Gertrudix *et al.*, (2024c).

## Results

### Objects of study and topics

The analysis of the objects of study and the theme of the studies (R.Q.1.1) allows us to group them into three major blocks, depending on how they approach the object of study. First, a considerable number of studies are interested in characterizing the personal characteristics and situations that encourage the spread of misinformation. Several studies focus on how certain individual characteristics such as gullibility, susceptibility to misinformation ("susceptibility to nonsense") or physiological conditions such as pregnancy may influence the propensity to believe and spread misinformation about climate change. In the same block, others are interested in understanding whether susceptibility to misinformation is influenced by the contexts in which it occurs. Different areas such as climate change, COVID-19 or the emergence of artificial intelligence are considered comparatively. Another large number of articles deal with the investigation of methods for detecting and combating misinformation, both with technological and methodological approaches. Interestingly, in most cases, the technological approach prevails through the application of automatic detection techniques using language models and machine learning and, in a smaller number of articles, fact-checking initiatives. Finally, a third group of articles analyses the impact of disinformation and misinformation on public and political perceptions.

### Theories that make up the theoretical framework from which the studies are approached

The theories (R.Q.1.2) explicitly referred to in the articles come mainly from the fields of psychology, sociology, communication and, to a lesser extent, international relations and criminology. The most frequently mentioned theory is inoculation theory ( McGuire, 1961a; McGuire, 1961b; McGuire, 1964) (n=9), followed by cognitive dissonance theory ( Festinger, 1957) (n=3) and evaluation theory (n=2). Other theories and analytical frameworks mentioned come from the field of cognitive and social psychology, including dual process theory ( Groves & Thompson, 1970), uncertainty reduction theory ( Berger & Calabrese, 1975), motivated reasoning theory ( Druckman & McGrath, 2019) ( Hart & Nisbet, 2012) ( Dawson *et al*., 2002); the theory of self-perception ( Bem, 1967), the theory of social judgment ( Sherif & Hovland, 1961), the theory of evaluation ( Arnold, 1960; Lazarus, 1966), the attribution theory of Heider (1958) and the theory of self-categorization of Turner *et al.*, (1987). Also worth mentioning are some theoretical approaches from the field of cognitive sociology such as social constructivism ( Berger & Luckmann, 1966). From the field of communication, the following should be emphasized: Media indexing theory ( Bennett, 1990), social communication theory ( Martín Serrano, 1986), agenda setting theory ( McCombs & Shaw, 1972), focused events theory ( Kingdon, 1984) and network gatekeeping theory ( Lewin, 1947; Lewin, 1951). Other outstanding contributions are the approaches originating from techno-deterministic theory such as the innovation system approach ( Malerba, 2004), the catch-up approach ( Abramovitz, 1986; Fagerberg, 1994), innovation learning systems ( Viotti, 2002), strategic narrative persuasion theory ( Ringsmose & Børgesen, 2011), warranting theory ( Walther & Parks, 2002), grounded theory ( Glaser & Strauss, 1999), theory of affect ( Tomkins, 1962) and ecological modernization theory ( Berger *et al.*, 2001; Fisher & Freudenburg, 2001; Foster, 2012).

### Methodologies used

A multivariate analysis was then conducted to characterize the studies according to the specific topics they address and the predominant methods they use (R.Q.1.2) in order to characterize the studies and determine the intensity (breadth and depth) of the phenomenon of climate change misinformation. To this purpose, the main and secondary taxonomy of the articles, the type of research, the hypotheses proposed, the study universe, the sampling method used, and the sample size are considered.

Considering the research focus, we can divide them into three categories: 1) research that focuses on the analysis of disinformation processes and dissemination strategies; 2) those that evaluate verification models and analyse the narratives for and against; and 3) studies that address how to counter climate change disinformation through education and communication. Compared to the topics of the established systemic misinformation matrix, these categories focus on concrete and procedural aspects or mitigation techniques rather than a global approach to the phenomenon.

### Data collection and analysis techniques

The studies analysed use heterogeneous data collection and analysis techniques (R.Q.2.2), which also reflects the diversity of epistemic and methodological approaches used. The complexity of the topic and the need to combine complementary fields and disciplines explain this diversity.

Data collection techniques focused on analysis account for 50% of the studies, with 23% using content analysis, 17% discourse analysis and 9% document analysis. The use of these techniques reflects an interest in examining the way misinformation about climate change is presented and communicated. Opinion polls (17.44%), interviews (12.79%) and experiments (10.47%) follow in second place. Observation techniques are used the least with 4.65%. The use of surveys and interviews indicates an interest in understanding attitudes, public perceptions and the reported impact of misinformation and, in some cases, assessing the effectiveness of communication strategies to combat misinformation.

In terms of analytical techniques, quantitative and qualitative approaches predominate, complemented by network analysis and mixed methods. Quantitative methods, especially those using multivariate analysis, are in the majority, accounting for around 40% of cases. The use of these methods demonstrates an interest in investigating relationships between multiple variables, either exploratory or confirmatory. Bivariate and univariate analyses are less common and account for about 15% of the methods used.

In 35% of cases, a qualitative analysis is carried out, using methods such as analytical induction, semiotic-structural analysis, grounded theory and content analysis. These cases aim to understand the meanings, discourses and structures that underlie misinformation about sustainability and therefore correspond to research that provides findings on how these messages are constructed and interpreted in different contexts. Network analysis, which focuses on information dissemination, sentiment and opinion analysis, and text mining, accounts for about 10% of the contributions. Finally, there is a small percentage (around 5%) of studies that combine qualitative and quantitative methods.

Examining the relationship between data collection methods and analysis techniques reveals a significant relationship between them. For example, attitude surveys are usually analysed using multivariate quantitative techniques. Content analysis and group experiments are used for both qualitative and quantitative data. Interviews and documentary analyses, which are primarily associated with inductive qualitative methods, are used to analyse discourses and narratives in articles that seek to understand how disinformation narratives are constructed and perceived.

### Nature of conclusions

The analysis of the conclusions (R.Q.3.1) reached by the studies analysed can be divided into those that explain the conditioning factors for the processing of information about climate change and the impact of disinformation about climate change on the public.

***Conditioning factors that modulate the processing of climate change information***

In terms of factors influencing the public's propensity to be influenced by misinformation about climate change and the impact of interventions aimed at creating positive attitudes towards climate science, the studies reviewed primarily point to the importance of experiential context as a determinant ( Benegal & Scruggs, 2018; Gruener, 2022; Sambrook *et al.*, 2021; Schmid-Petri & Bürger, 2022), cognitive factors related to information processing ( Zhou & Shen, 2021), the level of existing knowledge about climate change ( Lutzke *et al.*, 2019), the choice of topics addressed ( Gruener, 2022) and the perception of risk associated with the impact and consequences of climate change ( Moscato & Valencia, 2023; Ruiu & Ragnedda, 2021; Sambrook *et al.*, 2021). According to Van der Linden *et al.*, (2017), partisan ideology ranks above motivated reasoning in media selection and content consumption, which is also related to political polarization ( Feldman *et al.*, 2012; Krosnick & MacInnis, 2015; Leiserowitz *et al.*, 2013; McCright *et al.*, 2015). Research by McBeth, Lybecker and Sargent (2022), in line with the results of Benegal and Scrugg (2018), questions the predictive power of political ideology for climate attitudes. Their study showed no strict correlation between conservative political identity and climate skepticism and found evidence that individuals can be influenced by the narrator's identity through empathy, regardless of their ideological attribution.

For their part, Lutzke *et al.*, (2019) point to the low level of climate-specific knowledge as a primary obstacle to climate change mitigation measures. Gruener (2022) emphasizes the importance of the topics addressed in the content over other factors such as trust in social networks, the degree of perceived risk, the evidence contained in the messages or the way our thinking system adjusts to the information we receive. Lai *et al.*, (2022) link the presence of earlier physiological processes such as gestation to greater susceptibility to climate change disinformation and willingness to share it. 

On the other hand, the temporal and spatial proximity of the meteorological events and their severity also influence the impact of the measures taken. The notion of human catastrophe in the context of climate change distances the understanding of its impact from the individual realities of the public ( Moscato & Valencia, 2023). The adverse effects are presented in relation to unknown people and distant regions ( Ruiu & Ragnedda, 2021). This leads to a global dilution of the problem and limits its impact to the audience, which is free from all kinds of emotional involvement and individual responsibility. Sambrook *et al.*, (2021) argue, based on behavioural decision research, that extreme weather events only have an impact on judgments and decisions if they have occurred recently, and always depending on how strong the subjects' pre-existing beliefs about the reality of climate change are.

The extent of the perceived risk also affects the persuasiveness of the content. The evidence found by Dahlstrom and Rosenthal (2018) suggests that negative narratives about climate change are more likely to be ignored by audiences, while more neutral narratives have a greater chance of resonating with audiences because their impact is underestimated.

Other studies ( Zhou & Shen, 2021) confirmed that scientific evidence and misinformation can be processed differently. Thus, in the case of scientific evidence, the factuality of the message and the credibility of the source would influence attitudes after exposure to the message, in contrast to empathy, which would have no effect on scientific evidence. In contrast, perceived factuality of misinformation would have no effect on post-message attitudes, unlike empathy, where a direct effect would be found. These results would be explained by differences in the way scientific information is processed, which is more systematic, as opposed to false information, which is processed relatively superficially and automatically.

Malena-Chan (2019) presents the problems of inaction in the face of climate change because of the cognitive dissonance that subjects exhibited when confronted with narratives that encouraged involvement in environmental matters. Such an effect was present even among individuals with sufficient knowledge and personal motivation about climate change, due to emotional factors and moral contradictions they may experience at key moments of action (knowledge and understanding of climate change, agency and responsibility, and ability and action on the issue). 

***Effects of climate change-related misinformation on audiences***

Some studies showed contradictory results on the impact and consequences of public misinformation. The experiment conducted by Drummond *et al.*, (2020) found a moderate effect associated with exposure on three variables (belief in climate change, scientific consensus, and belief in scientists) and several covariates related to demographics and political ideology. While demographic characteristics and political ideology were stronger predictors than belief, the discreteness of the results allowed us to conclude that exposure to climate fake news is unlikely to strongly influence climate scepticism.

In contrast to the previous study, Wolff and Taddicken (2022) concluded that unbiased users are not completely resistant to the influence of climate change misinformation. A proactive stance in seeking information that confirms their initial position may explain this increased resistance. For their part, Van der Linden *et al.*, (2017) found that misinformation can cancel out the effectiveness of scientific consensus communication on climate change when both are presented together.

Koch *et al.* (2021) found evidence in an experiment conducted in Germany on the effectiveness of warning labels, which led them to conclude that people with left-wing ideology are more likely to be influenced and share misinformation. These findings contribute to the growing evidence for the effectiveness of cues, but at the same time question their relevance for systematic information processing versus motivated reasoning.

Lawrence and Estow (2017) found that individuals with a liberal ideology were more likely to react with frustration and respond with commentary to information opposing climate change. However, political orientation was not related to the tone of the comments, suggesting a degree of self-censorship among these individuals. The study also concluded that the preferred strategy was to use corrective information in comments rather than calling for collaboration. Conservatives were more likely to stick to information against climate change (backfire) than calls for collaboration in cases where the comment contained corrective information. It is therefore hypothesized that the call for collaboration may prove to be a more effective strategy as it invites dialog which allows for increased knowledge of the scientific consensus, which would impact pre-existing beliefs ( Goldberg *et al.*, 2019)

Freiling and Matthes (2023) found that user corrections were positively related to anger about climate change, which in turn was positively related to environmental activism. However, the data did not show a relationship between anger and correctness, nor between anger and environmental activism.

### Solutions and operational recommendations offered

The solutions and recommendations (R.Q.3.2) offered in the studies to combat climate change misinformation can be categorized into two groups: the effectiveness of fact-checking and audience inoculation strategies, and technological and methodological solutions for designing intervention models.

***Effectiveness of fact-checking and audience inoculation strategies***

Regarding the positive effects of fact-checking, Porter *et al.*, (2019) found evidence supporting the idea that party affiliation influences beliefs about climate change but is not determinant of their formation. For example, positive effects on corrective information were found for Trump's posted messages on climate change, although the influence was found to be greater for Democrats. These effects could not be replicated for messages related to environmental regulation. The results could find further explanation in the findings of Allen & McAleer (2018), who found a predominantly negative emotion in Trump's messages as well as a lack of a clear logical framework at the discursive level, suggesting which Trump is starting from a false understanding of climate change by confusing it with the concept of global warming.

Benegal and Scruggs (2018) challenge the premise of party affiliation as a predictor of climate change attitudes, finding evidence that bipartisan correctness leads Republicans to express much greater support for the scientific consensus on climate change. This evidence thus suggests that it is possible to narrow the gap between Republicans and Democrats.

Several studies looked at the benefits and limitations of vaccination as a solution to combat misinformation about climate change. Van der Linden *et al.*, (2017) found that the effectiveness of communication about the scientific consensus was nullified when presented in conjunction with misinformation. The authors saw positive effects in the use of vaccination messages to enhance the impact of scientific consensus messages. Subsequent studies ( Maertens *et al.*, 2020; Williams & Bond, 2020) have replicated earlier research findings, finding results that qualify or challenge the van der Linden *et al.*, (2017) findings. Maertens *et al.*, (2020) included a one-week period between vaccination and misinformation exposure in their design. The results suggest that the consensus message may be strong enough to remain significant on its own without the need for vaccination as additional protection, although differences are observed when followed by vaccination. In line with these results, it could be argued that since the consensus message is presented before the misinformation, the former could have a vaccine effect. In another replication of the same study, Williams and Bond (2020) found that providing information about the scientific consensus on climate change increased perceptions of the scientific consensus as much as inoculation, which occurred prior to the provision of misinformation. However, the authors were unable to replicate the finding that vaccination messages counteracted the effects of misinformation to a greater extent than the provision of information about the scientific consensus alone.

Other authors point out that the effectiveness of vaccination is related to the appropriateness of the messages for the context of the audience ( Green *et al.*, 2022; Lewandowsky, 2021; Treen *et al.*, 2020), which is why effective designs for vaccination education are needed. It is also hypothesized that the effectiveness of vaccination messages depends on the topics addressed and their potential impact on the target audiences ( Schmid-Petri & Bürger, 2022; Treen *et al.*, 2020). Based on these findings, further research would be needed to develop more effective vaccination education designs ( Green *et al.*, 2022).

***Technological and methodological solutions for the design of intervention models***

From the analysed selection, various studies deal with the possibilities offered by technologies in climate science communication. Authors such as Brannon *et al.*, (2021) emphasize the suitability of interactive journalistic products to present vaccination messages, and particularly the use of I-Docs together with gamification techniques. The application of computational methods in analysing the phenomenon of climate change communication would also have proven useful in identifying the institutional structures that fund and coordinate scientific misinformation campaigns.

The application of computational methods (natural language processing, machine learning and social network analysis) to the analysis of content has led to very interesting results. For example, research by Farrell *et al.*, (2019) and Coan *et al.*, (2021) clearly identified the financial and political roots on which denial discourses are based. Bhatia *et al.*, 2021 have applied supervised and semi-supervised computer models based on BERT to identify neutralization techniques used by climate change deniers.

Other work looks at the effectiveness of narratives to increase knowledge about climate change and counter misinformation. Some authors suggest the use of bottom-up models to demonstrate the impact of climate change on individual everyday experiences ( Howarth & Parsons, 2021; Ogra, 2022). Authors such as McQuaid *et al.*, (2018) conclude that citizen participation in policy development needs to be encouraged and that communication is more effective when it does not originate from the authorities ( Bevan *et al.*, 2020). These suggestions are also supported by the findings of studies examining climate change communication at the local level. Such studies point to the need to involve citizens in both the problem and the solution, moving away from their usual victim role, while increasing scientific knowledge about climate change ( Ejaz *et al.*, 2021; Naguimbing-Manlulu, 2021).

Other studies point to the ineffectiveness of narratives that exploit fear or offer an overly catastrophic view of the consequences of climate change. On this aspect, Segal (2017) suggests that utopian or dystopian narratives leave audiences stuck in the debate between extremes. Alternative models of narratives are proposed by Herr (2022), who points to a design based on the concept of ecological hope, as opposed to the dominant concept of fear. For their part, Choi and Leckie (2018) focus on exploring the slow causalities of geological and generational processes, for which there is little empirical evidence, and Largo Loayza (2022) focuses on the design of models of transmedia narratives and on the integration of storytelling.

***Local studies: media coverage of climate change and the spread of misinformation in social networks***

Of the sample analysed, a significant percentage of the articles (42.7% n=32) deal with the phenomenon from a local perspective. These are mainly qualitative studies dealing with the analysis of the narratives from a structural and thematic point of view, the actors involved (promoters and recipients) and the disinformation and fact-checking strategies. The selected sample allows an approach to the phenomenon from a broad geographical perspective. Most of the studies focus on the United States (n=11) and Canada (n=2), on European countries such as the United Kingdom (n=3), Germany (n=2), the Baltic countries (n=2) and Sweden (n=1), on BRICS countries such as Brazil (n=3), China (n=2) and India (n=2), and on other recently emerging countries such as Malaysia (n=1) or developing countries such as Pakistan (n=1) and Uganda (n=1).

Some of these studies (n=6) correspond to content analysis of news from local newspapers in the Philippines ( Naguimbing-Manlulu, 2021), Malaysia ( Hassan *et al.*, 2023), Pakistan ( Ejaz *et al.*, 2021), Latvia ( Kleinberga *et al.*, 2023), the USA and China ( Moscato & Valencia, 2023) and the UK ( Ruiu & Ragnedda, 2021). Other work draws on surveys (n=4) ( Chu *et al.*, 2023; Cia Alves *et al.*, 2022; Hong, 2020; Ogra, 2022; Ruiu & Ragnedda, 2021) or interviews (n=1) ( McQuaid *et al.*, 2018) to identify how citizens perceive the problem, or website and social network analysis ( Al-Rawi *et al.*, 2021) to examine misinformation and its impact. There are also examples of pre-designed experiments ( de Almeida Soares & Gomes Barreto, 2022; Drummond *et al.*, 2020; Koch *et al.*, 2021; Lawrence & Estow, 2017; Vu *et al.*, 2022) that look at the impact of misinformation and the effectiveness of remedies. Finally, other papers (n=2) aim to debunk models of narratives.

The media is often portrayed as amplifying the discourse of political elites in relation to climate change ( Ejaz *et al.*, 2021; Hassan *et al.*, 2023; Kleinberga *et al.*, 2023; Naguimbing-Manlulu, 2021; Silva, 2022), with the patterns being the same across countries ( Dunwoody & Peters, 1992). Little or no attention is paid to the average citizen, who is not seen as part of the problem, and in some cases is portrayed in a victim role ( Naguimbing-Manlulu, 2021). Similarly, there is a tendency to present climate change in a global dimension without addressing its local impacts ( McQuaid *et al.*, 2018), nor the complexity of mitigation and adaptation approaches ( Kleinberga *et al.*, 2023). In this sense, climate reporting has gone beyond the norm of balance to become interpretive journalism, as Brüggemann and Engesser (2017) have noted. Also common is the lack of scientific explanations or individually implementable solutions ( Ejaz *et al.*, 2021; Naguimbing-Manlulu, 2021), leading to a lack of citizen engagement.

This discursive monopoly would also enable the spread of disinformation ( Allen & McAleer, 2018; Hassan *et al.*, 2023; Porter *et al.*, 2019; Silva, 2022) embedded in political propaganda, with the case of Bolsonaro in Brazil or Trump in the US being clear representatives of this strategy ( Silva, 2022).

Based on the topics addressed in the climate change content reviews, the main areas of disinformation can be identified.

## Discussion

The analysis of the objects of study, topics and theories under which the object of study is treated reflects a trend towards a multidimensional understanding of the problem. However, although recent studies deal with a variety of objects and topics and draw on a wide range of theories, there is a notable disconnect from the financial, economic and political roots of disinformation.

Findings suggest that despite interest in understanding the personal and contextual dynamics that foster climate disinformation ( Benegal & Scruggs, 2018; Sambrook *et al.*, 2021; Schmid-Petri & Bürger, 2022), and efforts to develop technologies and methods to detect and neutralize it ( Gruener, 2022; Lutzke *et al.*, 2019), there is a limitation in looking at the phenomenon from a systemic perspective that includes financial, economic and political dimensions, as documented in previous studies on the influence of the hydrocarbon industry on the development of climate disinformation ( Oreskes & Conway, 2011; Supran & Oreskes, 2017). This fragmented approach contrasts with evidence showing that disinformation was a coordinated strategy used by actors with specific economic and political interests to influence public perceptions and policies on climate change ( Gelbspan, 1997; Gore, 1993). In this sense, the literature has emphasized the importance of the financial, economic and political roots behind climate obstructionism ( Almiron & Moreno, 2022; Dunlap & McCright, 2011), suggesting that an effective understanding of the phenomenon requires an analysis that integrates these dimensions in a systemic manner.

This confirms the relevance of hypothesis 1 and underlines the need for more integrative research approaches that not only address the technological and methodological aspects of climate misinformation, but also consider the power and funding structures that support and promote these narratives. Thus, by focusing on concrete and procedural aspects or mitigation techniques rather than global approaches, there is still a significant gap in the systematic and global coverage of the topic. Therefore, **H1 is only partially confirmed**, suggesting that systemic analysis needs to be further integrated into climate disinformation research. In this sense, studies should be developed that bring together methodological advances in the detection of misinformation with a critical analysis of the economic and political forces that perpetuate it, as suggested by the findings of Farrell *et al.* (2019) and the proposals for strategies to combat scientific misinformation by Oreskes & Conway (2011) and Supran & Oreskes (2017).

In terms of research methods and techniques, they show that although a diversity of methods and techniques for data collection and analysis can be observed in the recent literature, the ability of these investigations to translate into operational solutions and recommendations still offers significant scientific challenges.

The studies examined use a variety of methodological approaches, including content and discourse analysis, surveys, and experiments ( Cook *et al.*, 2015; Farrell *et al.*, 2019). This diversity of methods reflects the complexity of the phenomenon of climate misinformation and the need for multidisciplinary approaches to capture its various dimensions. However, the impact of these methods on the development of effective operational solutions is limited. In any case, the challenges in combating climate disinformation are not only epistemological, but also structural and political. Previous literature has emphasized the importance of understanding disinformation as part of a broader obstructionist strategy funded and promoted by specific economic and geopolitical interests ( Oreskes & Conway, 2011; Supran & Oreskes, 2017). Therefore, the methods used need to be broadened to include critical analyses of the power and funding systems that support climate disinformation. This involves not only identifying disinformation tactics, but also the actors, networks and resources that facilitate them. These aspects have been emphasized in works such as those by Dunlap and McCright (2011) and Almiron & Moreno (2022).

The variety of methods and data collection and analysis techniques used in the research reflects the effort to adapt the study methods to the complexity of the phenomenon of climate misinformation. However, the relationship between the choice of methods and the ability to identify and propose effective solutions is not explicitly analysed in the results presented. **H2 is only partially confirmed**, showing that despite methodological efforts in this direction, the effectiveness of research in translating its contributions into concrete and effective operational solutions is not uniform.

Finally, analysing the findings and the solutions and recommendations from the perspective that it is important to consider climate disinformation not only as an isolated phenomenon, but as part of a broader structure of climate obstructionism underpinned by financial, economic and political roots, shows that obstructionism in relation to climate action is deeply rooted in economic and political interests aimed at maintaining the status quo ( Oreskes & Conway, 2011; Supran & Oreskes, 2017). The effectiveness of interventions against climate misinformation therefore depends on the ability of research to understand and address these underlying dynamics and whether it can demonstrate this systemic understanding of the phenomenon. This is supported by recent research that points to the need to implement communication and education strategies that not only warn against misinformation and disinformation, but also build citizen resilience ( Cook *et al.*, 2015; van der Linden *et al.*, 2017). However, there is a significant gap in the existing literature, which lacks more comprehensive approaches that combine critical analyses of the financial, economic and political roots of misinformation with concrete proposals for action. Although some studies have begun to explore these relationships, there is still a lack of greater inclusion of interdisciplinary analyses that can provide a complete picture of how climate disinformation is produced and disseminated and how it can be effectively combated.

In this sense, despite considerable progress, the ability to provide operational recommendations that encompass the complexity of climate disinformation is limited, so **H3 is only partially confirmed**, as more systematized approaches are needed that can effectively influence policies and practice against climate disinformation.

## Conclusions

The following conclusions emerge from the discussion of the results, focusing both on the progress identified and, on the limitations, and gaps in research on climate change-related misinformation from the perspective of obstructionism:

1. **Limitations in the collection of scientific literature.** The studies analysed are limited as they mainly deal with local studies on narratives and the impact of disinformation, vaccination and fact-checking at the local level. This has implications for the information they provide and, more importantly, their ability to provide knowledge that can be used for decision-making. There is a lack of studies dealing with, for example, comparative perspectives, policy analysis or the assessment of groups with attitudes, especially those that play an important role in delaying or preventing climate action. This limitation, which conditions the availability of a broader and more systemic view of the phenomenon, not only leads studies to focus their analysis on less valuable and urgent aspects, but also favours the use of disinformation as a strategy to obstruct climate action, as the variables that influence or influence the effectiveness of anti-disinformation measures are not revealed, preventing political and practical action to combat it.

2. **Thematic and theoretical diversity and diversity of approaches to the object of study.** The variety of approaches to the object of study shows the complexity of its approach, but also the difficulty of conducting more systemic and global analyses. Although some of the studies are interested in identifying the personal and contextual factors that promote climate disinformation, in most cases this is done from very specific and limited perspectives. Assessing the development of technological tools to detect and neutralize it and analysing its impact on public and political perceptions are valuable in terms of intervention techniques, but do not help to understand the core of the components that make up the matrix of systemic disinformation with financial, economic and political roots proposed in this study.

This disordered plurality is also reflected in the wide range of theories and analytical frameworks used, which come from psychology, sociology, communication, international relations or criminology and highlight the theory of inoculation, cognitive dissonance and judgment theory.

3. **More systematized methodological approaches are needed.** The diversity of methodological approaches and the lack of large-scale studies limit the development of replication studies that could help increase the type and number of interventions and measure their effectiveness. Consolidation and systematization of methodological study models would help to simplify study design, expand samples, and harmonize data collection and analysis techniques, which would strengthen research and facilitate the development of meta-analyses.

4. **Large-scale research should be funded.** The limitations of the sample designs also show that in most cases these are well-designed studies, but small in scale and scope. To bridge the gap between micro and macro research, funding lines and calls for proposals should be established to promote the development of large-scale research, such as studies covering all countries of the European Union. This global approach would facilitate the development of more integrated approaches that identify, based on a critical analysis, the fundamental elements that shape the landscape of scientific misinformation, focusing on the practices used as obstructionist tactics against climate change action.

5.      It is necessary to **promote studies that analyse the systemic nature of the disinformation of the financial, economic and political climate**. This need is reflected in the conclusions of the studies, which only partially explain the components of the matrix of systemic disinformation with financial, economic and political roots. The studies describe and document the factors that influence the processing of information about climate change and the impact of disinformation on audiences. This allows us to understand some of the psychological and social dynamics that facilitate the spread of disinformation, but they deal only to a limited extent with specific mechanisms such as critical scientific scrutiny, denial campaigns or the faking of scientific uncertainty.

The same applies to the studies that explain how individual predispositions and risk perception influence susceptibility to climate change information. However, they do not address how these strategies can be part of coordinated obstructionist campaigns, nor do they explain the extent to which critical scientific reviews, which have been documented as essential in the processes of scientific discrediting and confusion, can be systematically deployed along with strategic sponsorship actions by entities with a specific interest in delaying or postponing climate action, policies, or programs.

6. **Research should provide solutions or operational recommendations.** The effectiveness of fact-checking and inoculation strategies, as well as technological and methodological solutions for developing intervention models, represent significant advances in the fight against misinformation. However, these solutions focus primarily on target audiences and the detection or correction of misinformation without addressing in depth the underlying mechanisms and complex strategies that characterize systemic disinformation promoted by actors with specific interests.

Moreover, while these techniques can mitigate the impact of disinformation on individuals and strengthen their resilience to information manipulation, in their formalized model they are ill-equipped to respond to the hidden structures and funding of large-scale disinformation campaigns which rely on components such as critical scientific reviews, denial campaigns or the fabrication of scientific uncertainty, which in many cases are only discovered after they have already developed and taken effect.

The study provides valuable results to understand and defuse scientific misinformation. However, as it does not focus on the networks of influence promoted by financial actors, politicians and third countries, it does not offer operational solutions to intervene in a space of information pollution whose understanding goes beyond the simple dissemination of false information about climate change, but rather functions in terms of a complex structural and systemic set-up in which misinformation is key to the success of climate mitigation policies.

## Data Availability

NO data are associated with this article. Zenodo Sample Records: Disinformation as a strategy of obstructionism on climate action: analysis of the limitations of the scientific literature for a systemic understanding of the phenomenon. https://doi.org/10.5281/zenodo.10640341. ( Gertrudix *et al.*, 2024a). The project contains the following underlying data: 01.1_PRIMERPRISMA_IDENTIFICATION.xlsx: First, 6 general terms from the area of environment and sustainability and 11 specific terms from the area of disinformation were selected. This file summarizes the selected keywords, the generated Boolean operators, and the initial search results, retrieving 783 records. 01.2_PRIMER PRIMA-SCREENING.xlsx: Details the screening process, eliminating duplicates and non-English documents, resulting in 271 retained records. 01.3_PRIMER PRISMA_INCLUDED.xlsx: Contains the results after further screening, with 82 documents retained and expanded data including bibliometric details. 02.1_SEGUNDOPRISMA_IDENTIFICATION.xlsx: Covers the second phase of identification using new terms related to climate and disinformation, retrieving 174 records. 02.2_SEGUNDOPRISMA_SCREENING.xlsx: Includes the screening process for the second phase, reducing records to 75, with an abstract review retaining 2 documents. 02.3_SEGUNDOPRISMA_INCLUDED.xlsx: Integrates documents from both search phases and other sources, culminating in a final review of 86 documents. 3.1_Other sources.xlsx: Includes additional relevant sources identified during the review process. 4-Final included.xlsx: Contains the final set of 75 publications subjected to the DESLOCIS analysis model. Data are available under the terms of the Creative Commons Attribution 4.0 International license (CC-BY 4.0). Sample Records (Analytical procedure): Disinformation as a strategy of obstructionism on climate action: analysis of the limitations of the scientific literature for a systemic understanding of the phenomenon. Zenodo. https://doi.org/10.5281/zenodo.12722934 The project contains the following underlying data: Formulario de análisis SLR 5-Datos forms-original Sample Records ( PRISMA Checklist and Flow diagram): Disinformation as a strategy of obstructionism on climate action: analysis of the limitations of the scientific literature for a systemic understanding of the phenomenon. https://doi.org/10.5281/zenodo.12722440 ( Gertrudix *et al.*, 2024b). Data are available under the terms of the Creative Commons Attribution 4.0 International license (CC-BY 4.0).

## Software availability statement

While Publish or Perish is free to use, it is not open source. As an alternative, Zotero provides equivalent functionalities and is fully open access.

## References

[ref-1] Abramovitz M : Catching up forging ahead, and falling behind. *J Econ Hist.* 1986;46(2):385–406. 10.1017/S0022050700046209

[ref-2] Abudu H Wesseh PK Jr Lin B : Does political propaganda matter in climate change? Insights from the United States of America. *Journal of Management Science and Engineering.* 2023;8(3):386–397. 10.1016/j.jmse.2022.12.006

[ref-3] Allen DE McAleer M : Fake news and indifference to scientific fact: President Trump’s confused tweets on global warming, climate change and weather. *Scientometrics.* 2018;117:625–629. 10.1007/s11192-018-2847-y

[ref-4] Almiron N Moreno JA : Más allá del negacionismo del cambio climático. Retos conceptuales al comunicar la obstrucción de la acción climática. *Ámbitos.Revista Internacional De Comunicación.* 2022; (55):9–23. 10.12795/Ambitos.2022.i55.01

[ref-5] Al-Rawi A OʼKeefe D Kane O : Twitter’s Fake News Discourses Around Climate Change and Global Warming. *Frontiers in Communication, Sec. Science and Environmental Communication.* 2021;6. 10.3389/fcomm.2021.729818

[ref-6] Arnold MB : Emotion and personality.Columbia University Press,1960;1. Reference Source

[ref-7] Bem DJ : Self-perception: an alternative interpretation of cognitive dissonance phenomena. *Psychol Rev.* 1967;74(3):183–200. 10.1037/h0024835 5342882

[ref-8] Benegal SD Scruggs LA : Correcting misinformation about climate change: the impact of partisanship in an experimental setting. *Clim Change.* 2018;148(1–2):61–80. 10.1007/s10584-018-2192-4

[ref-9] Bennett L : Toward a theory of press-state relations in the united states. *J Commun.* 1990;40(2):103–127. 10.1111/j.1460-2466.1990.tb02265.x

[ref-10] Berger CR Calabrese RJ : Some explorations in initial interaction and beyond: toward a developmental theory of interpersonal communication. *Hum Commun Res.* 1975;1(2):99–112. 10.1111/j.1468-2958.1975.tb00258.x

[ref-11] Berger G Flynn A Hines F : Ecological modernization as a basis for environmental policy: Current environmental discourse and policy and the implications on environmental supply chain management. *Innovation: The European Journal of Social Science Research.* 2001;14(1):55–72. 10.1080/13511610125074

[ref-12] Berger P Luckmann T : The social construction of reality: a treatise in the sociology of knowledge.Anchor Books,1966. Reference Source

[ref-13] Bevan LD Colley T Workman M : Climate change strategic narratives in the United Kingdom: emergency, extinction, effectiveness. *Energy research & social science.* 2020;69: 101580. 10.1016/j.erss.2020.101580

[ref-14] Bhatia S Lau JH Baldwin T : Automatic classification of neutralization techniques in the narrative of climate change scepticism. *Proceedings of the 2021 Conference of the North American Chapter of the Association for Computational Linguistics: Human Language Technologies.* 2021;2167–2175. 10.18653/v1/2021.naacl-main.175

[ref-15] Bilott R : Exposure: Poisoned water, corporate greed, and one lawyer's twenty-year battle against DuPont.Atria Books,2020. Reference Source

[ref-16] Bovet A Makse HA : Influence of fake news in twitter during the 2016 US presidential election. *Nat Commun.* 2019;10(1): 7. 10.1038/s41467-018-07761-2 30602729 PMC6315042

[ref-17] Brannon L Gold L Magee J : The potential of interactivity and gamification within immersive journalism & Interactive Documentary (I-Docs) to explore climate change literacy and inoculate against misinformation. *Journalism Practice.* 2021;16(2–3):334–364. 10.1080/17512786.2021.1991439

[ref-18] Brüggemann M Engesser S : Beyond false balance: how interpretive journalism shapes media coverage of climate change. *Glob Environ Change.* 2017;42:58–67. 10.1016/j.gloenvcha.2016.11.004

[ref-19] Carbonell-Alcocer A Romero-Luis J Gertrudix M : A methodological assessment based on a systematic review of circular economy and bioenergy addressed by education and communication. *Sustainability.* 2021;13(8): 4273. 10.3390/su13084273

[ref-20] Carbonell-Alcocer A Romero-Luis J Gertrudix M : Educating for a sustainable future through the circular economy: citizen involvement and social change. *Comunicar.* 2022;30(73):21–32. 10.3916/C73-2022-02

[ref-21] Carbonell-Alcocer A Romero-Luis J Gertrudix M : Datasets on the assessment of the scientific publication's corpora in circular economy and bioenergy approached from education and communication. *Data Brief.* 2023;47: 108958. 10.1016/j.dib.2023.108958 36879608 PMC9984419

[ref-22] Castoriadis C : L'institution imaginaire de la société.Seuil,1975. Reference Source

[ref-23] Chen CF Shi W Yang J : Social bots’ role in climate change discussion on Twitter: measuring standpoints, topics, and interaction strategies. *Advances in Climate Change Research.* 2021;12(6):913–923. 10.1016/j.accre.2021.09.011

[ref-24] Choi TY Leckie B : Slow causality: the function of narrative in an age of climate change. *Vic Stud.* 2018;60(4):565–587. 10.2979/victorianstudies.60.4.03

[ref-25] Chu J Zhu Y Ji J : Characterizing the semantic features of climate change misinformation on Chinese social media. *Public Underst Sci.* 2023;32(7):845–859. 10.1177/09636625231166542 37162274

[ref-26] Cia Alves EE de Albuquerque RB Ferreira MA : Do non-state actors influence climate change policy? Evidence from the Brazilian nationally determined contributions for COP21. *Journal of Politics in Latin America.* 2022;14(1):120–140. 10.1177/1866802X211034187

[ref-27] Coan T Boussalis C Cook J : Computer-assisted detection and classification of misinformation about climate change. *Sci Rep.* 2021;11: 22320. 10.31235/osf.io/crxfm 34785707 PMC8595491

[ref-28] Cook J Ecker U Lewandowsky S : Misinformation and how to correct it. *Emerging Trends in the Social and Behavioral Sciences: An Interdisciplinary, Searchable, and Linkable Resource.* 2015;1–17. 10.1002/9781118900772.etrds0222

[ref-29] Cook J Supran G Oreskes N : Exxon has misled Americans on climate change for decades. Here’s how to fight back. *The Guardian.* (2019, 23/10). Reference Source

[ref-30] Dawson E Gilovich T Regan DT : Motivated reasoning and performance on the was on selection task. *Pers Soc Psychol Bull.* 2002;28(10):1379–1387. 10.1177/014616702236869

[ref-31] Dahlstrom MF Rosenthal S : Third-person perception of science narratives: the case of climate change denial. *Sci Commun.* 2018;40(3):340–365. 10.1177/1075547018766556

[ref-142] de Almeida Soares AM Gomes Barreto C : Disputes and narratives on the distributed generation of electricity in Brazil: setbacks for the 2030 Agenda for sustainable development and the Paris agreement. *Sustainability in Debate.* 2022;13(3):32–71. 10.18472/SustDeb.v13n3.2022.45621

[ref-32] Denzin NK : Sociological methods: a sourcebook. Routledge,2017. Reference Source

[ref-33] Druckman JN McGrath MC : The evidence for motivated reasoning in climate change preference formation. *Nat Clim Change.* 2019;9(2):111–119. 10.1038/s41558-018-0360-1

[ref-34] Drummond C Siegrist M Árvai J : Limited effects of exposure to fake news about climate change. *Environ Res Commun.* 2020;2(8): 081003. 10.1088/2515-7620/abae77

[ref-35] Dunlap RE McCright AM : Climate change denial: sources, actors, and strategies. In: C. Lever-Tracy (Ed.) *Routledge handbook of climate change and society.* Routledge Press,2010;240–259. Reference Source

[ref-36] Dunlap RE McCright AM : Organized climate change denial.In: J. S. Dryzek, R. B. Norgaard & D. Schlosberg (Eds.), *The Oxford Handbook of Climate Change and Society.*Oxford University Press,2011;144–160. 10.1093/oxfordhb/9780199566600.003.0010

[ref-37] Dunwoody S Peters HP : Mass media coverage of technological and environmental risks: a survey of research in the United States and Germany. *Public Underst Sci.* 1992;1(2):199–230. 10.1088/0963-6625/1/2/004

[ref-38] eComciencia: Nuevas narrativas interactivas e inmersivas para impulsar la economía circular y la innovación social a través de la comunicación científica y la ciencia ciudadana desde la escuela proyecto.PID2021-127019OB-I00, Ciberimaginario,2023. Reference Source

[ref-40] Ehrlich PR Wolff G Daily GC : Knowledge and the environment. *Ecol Econ.* 1999;30(2):267–284. 10.1016/S0921-8009(98)00130-X

[ref-39] Ejaz W Ittefaq M Arif M : Understanding influences, misinformation, and fact-checking concerning climate-change journalism in Pakistan. *Journalism Practice.* 2021;16(2–3):404–424. 10.1080/17512786.2021.1972029

[ref-41] European Commission: Global climate action Agenda.Climate Action,2022.

[ref-42] European Commission: The European green deal: striving to be the first climate-neutral continent. 2023. Reference Source

[ref-43] Fagerberg J : Technology and international differences in growth rates. *J Econ Lit.* 1994;32(3):1147–1175. Reference Source

[ref-44] Farrell J : Corporate funding and ideological polarization about climate change. *Proc Natl Acad Sci U S A.* 2016;113(1):92–97. 10.1073/pnas.1509433112 26598653 PMC4711825

[ref-45] Farrell J McConnell K Brulle R : Evidence-based strategies to combat scientific misinformation. *Nat Clim Chang.* 2019;9(3):191–195. 10.1038/s41558-018-0368-6

[ref-46] Feldman L Maibach EW Roser-Renouf C : Climate on cable: the nature and impact of global warming coverage on Fox News, CNN, and MSNBC. *Int J Press Polit.* 2012;17(1):3–31. 10.1177/1940161211425410

[ref-47] Fernández-Castrillo C Magallón-Rosa R : El periodismo especializado ante el obstruccionismo climático. El caso de Maldito Clima. *Revista Mediterránea De Comunicación.* 2023;14(2):35–52. 10.14198/MEDCOM.24101

[ref-48] Festinger L : A theory of cognitive dissonance.Stanford University Press,1957. Reference Source

[ref-49] Fisher DR Freudenburg WR : Ecological modernization and its critics: assessing the past and looking toward the future. *Soc Nat Resour.* 2001;14(8):701–709. 10.1080/08941920119315

[ref-50] Foster JB : The planetary rift and the new human exemptionalism: a political-economic critique of ecological modernization theory. *Organ Environ.* 2012;25(3):211–237. 10.1177/1086026612459964

[ref-51] Freiling I Matthes J : Correcting climate change misinformation on social media: reciprocal relationships between correcting others, anger, and environmental activism. *Comput Hum Behav.* 2023;145: 107769. 10.1016/j.chb.2023.107769

[ref-53] Gelbspan R : The heat is on: the high stakes battle over Earth's threatened climate.Addison-Wesley Pub. Co.,1997. Reference Source

[ref-55] Gertrudix M Carbonell-Alcocer A Arcos R : Sample records: disinformation as a strategy of obstructionism on climate action: analysis of the limitations of the scientific literature for a systemic understanding of the phenomenon. (Versión 1). Zenodo. 2024a. 10.5281/zenodo.10640341 PMC11467642 39399659

[ref-56] Gertrudix M Carbonell-Alcocer A Arcos R : Sample records (PRISMA Checklist and flow diagram): disinformation as a strategy of obstructionism on climate action: analysis of the limitations of the scientific literature for a systemic understanding of the phenomenon. (Versión 1). Zenodo. 2024b. 10.5281/zenodo.12722440

[ref-57] Gertrudix M Carbonell-Alcocer A Arcos R : Sample records (analytical procedure): disinformation as a strategy of obstructionism on climate action: analysis of the limitations of the scientific literature for a systemic understanding of the phenomenon. (Versión 1). Zenodo. 2024c. 10.5281/zenodo.12722934

[ref-54] Gertrudix M Romero-Luis J Carbonell-Alcocer A : Descriptors for a systematic literature review on social sciences (DESLOCIS). (Versión 1). *Zenodo.* 2021. 10.5281/zenodo.4462764

[ref-58] Glaser B Strauss A : The discovery of grounded theory: strategies for qualitative research (1st ed.).Routledge,1999. 10.4324/9780203793206

[ref-59] Glenza J : Revealed: the free-market groups helping the tobacco industry. *The Guardian.* 2019. Reference Source

[ref-170] Goldberg MH van der Linden S Ballew MT : The role of anchoring in judgments about expert consensus. *J Appl Soc Psychol.* 2019;49(5):349–362. 10.1111/jasp.12576

[ref-60] Goldman GT Berman E Halpern M : Ensuring scientific integrity in the age of trump. *Science.* 2017;355(6326):696–698. 10.1126/science.aam5733 28209862

[ref-61] González CA Agudo A : The tobacco industry and the manipulation of scientific investigation. the case of European study of IARC-WHO related to tobacco passive consumption and lung cancer. *Med Clin (Barc).* 2000;115(8):302–304. 10.1016/s0025-7753(00)71541-7 11093886

[ref-62] Gordon A Suzuki DT : It's a matter of survival.Harvard University Press,1991. Reference Source

[ref-63] Gore A : Earth in the balance: ecology and the human spirit. *J Leis Res.* 1993;25(2):218.

[ref-64] Gramling C : Climate change disinformation is evolving. so are efforts to fight back. *Sci News.* 2021. Reference Source

[ref-65] Green M McShane CJ Swinbourne A : Active versus passive: evaluating the effectiveness of inoculation techniques in relation to misinformation about climate change. *Aust J Psychol.* 2022;74(1): 2113340. 10.1080/00049530.2022.2113340

[ref-66] Groves PM Thompson RF : Habituation: a dual-process theory. *Psychol Rev.* 1970;77(5):419–450. 10.1037/h0029810 4319167

[ref-67] Gruener S : Determinants of gullibility to misinformation: a study of climate change, COVID-19 and Artificial Intelligence. *Journal of Interdisciplinary Economics.* 2022;36(1):58–78. 10.1177/02601079221083482

[ref-68] Hafezi M Sahin O Stewart RA : Creating a novel multi-layered integrative climate change adaptation planning approach using a systematic literature review. *Sustainability.* 2018;10(11): 4100. 10.3390/su10114100

[ref-69] Hahn RW : Toward a new environmental paradigm. *Yale Law J.* 1993;102:1719–1761. 10.2307/796830

[ref-70] Haltinner K Sarathchandra D : Inside the world of climate change skeptics.University of Washington Press,2023. Reference Source

[ref-72] Hart PS Nisbet EC : Boomerang effects in science communication: How motivated reasoning and identity cues amplify opinion polarization about climate mitigation policies. *Commun Res.* 2012;39(6):701–723. 10.1177/0093650211416646

[ref-71] Harzing AW : Publish or Perish. 2007. Reference Source

[ref-73] Hassan I Musa RM Latiff Azmi MN : Analysis of climate change disinformation across types, agents and media platforms. *Inform Dev.* 2023. 10.1177/02666669221148693

[ref-74] Heider F : The psychology of interpersonal relations.John Wiley & Sons Inc,1958. 10.1037/10628-000

[ref-75] Herr A : Narratives of hope: imagination and alternative futures in climate change literature. *Transcience.* 2022;13(2):88–111. Reference Source

[ref-76] Herranen O : Understanding and overcoming climate obstruction. *Nat Clim Chang.* 2023;13(6):500–501. 10.1038/s41558-023-01685-6

[ref-77] Homar AR Cvelbar LK : The effects of framing on environmental decisions: a systematic literature review. *Ecol Econ.* 2021;183: 106950. 10.1016/j.ecolecon.2021.106950

[ref-78] Hong SC : Presumed effects of “fake news” on the global warming discussion in a cross-cultural context. *Sustainability.* 2020;12(5): 2123. 10.3390/su12052123

[ref-79] Horner C : Red hot lies: How global warming alarmists use threats, fraud, and deception to keep you misinformed.Washignton DC: Regnery Publishing Inc,2008. Reference Source

[ref-80] Hornsey MJ Fielding KS : Understanding (and reducing) inaction on climate change. *Soc Issues Policy Rev.* 2020;14(1):3–35. 10.1111/sipr.12058

[ref-81] Howarth C Parsons L : Assembling a coalition of climate change narratives on UK climate action: a focus on the city, countryside, community and home. *Clim Change.* 2021;164(1–2): 8. 10.1007/s10584-021-02959-8

[ref-82] IPCC: AR6 Synthesis Report: Climate Change 2023. 2023. Reference Source

[ref-83] Jacques PJ Dunlap RE Freeman M : The organisation of denial: Conservative think tanks and environmental scepticism. *Environ Polit.* 2008;17(3):349–385. 10.1080/09644010802055576

[ref-84] Kingdon JW : Agendas, alternatives, and public policies.Cambridge University Press,1984. Reference Source

[ref-85] Kleinberga V Palkova A Dace E : How to recognise the inevitable: Latvian media narratives on climate change. *Environ Dev.* 2023;45: 100816. 10.1016/j.envdev.2023.100816

[ref-87] Koch T Frischlich L Lermer E : The effects of warning labels and social endorsement cues on credibility perceptions of and engagement intentions with fake news. *J Appl Soc Psychol.* 2021;1(13). 10.31234/osf.io/fw3zq

[ref-86] Krosnick J MacInnis B : Fox and not-fox television news impact on opinions on global warming: selective exposure, not motivated reasoning.In: J. P. Forgas, K. Fiedler, W. D. Crano (Eds.), *Social Psychology and Politics.*Taylor and Francis,2015;75–90. 10.4324/9781315717104-11

[ref-88] Lai K Yang Y Na Y : The relationship between bullshit receptivity and willingness to share misinformation about climate change: the moderating role of pregnancy. *Int J Environ Res Public Health.* 2022;19(24): 16670. 10.3390/ijerph192416670 36554551 PMC9779392

[ref-89] Lakoff G : The political mind: A cognitive scientist's guide to your brain and its politics.Penguin,2008. Reference Source

[ref-90] Largo Loayza J : Narrativas transmedia para concientizar sobre la contaminación minera de ríos del ecuador *a review for the environmental awareness*. * International Visual Culture Review / Revista Internacional de Cultura Visual.* 2022;10(1):1–8. 10.37467/revvisual.v9.3566

[ref-91] Larson EV Darilek RE Gibran D : Foundations of effective influence operations: a framework for enhancing army capabilities.Arroyo Center,2009. Reference Source

[ref-93] Lawrence EK Estow S : Responding to misinformation about climate change. *Appl Environ Educ Commun.* 2017;16(2):117–128. 10.1080/1533015X.2017.1305920

[ref-92] Lazarus RS : Psychological stress and the coping process.McGraw-Hill,1966. Reference Source

[ref-95] Leiserowitz A Maibach E Rosenthal S : Climate change in the American mind: beliefs & attitudes.Yale University and George Mason University, New Haven, CT: Yale Program on Climate Change Communication,2023. Reference Source

[ref-94] Leiserowitz AA Maibach EW Roser-Renouf C : Climategate, public opinion, and the loss of trust. *Am Behav Sci.* 2013;57(6):818–837. 10.1177/0002764212458272

[ref-96] Lewandowsky S : Climate change disinformation and how to combat it. *Annu Rev Public Health.* 2021;42:1–21. 10.1146/annurev-publhealth-090419-102409 33355475

[ref-97] Lewin K : Frontiers in group dynamics: II. Channels of group life; social planning and action research. *Human Relations.* 1947;1(2):143–153. 10.1177/001872674700100201

[ref-98] Lewin K : Field theory in social science: selected theoretical papers.(Edited by Dorwin Cartwright), 1951. Reference Source

[ref-99] Lutzke L Drummond C Slovic P : Priming critical thinking: simple interventions limit the influence of fake news about climate change on Facebook. *Glob Environ Change.* 2019;58: 101964. 10.1016/j.gloenvcha.2019.101964

[ref-100] Maertens R Anseel F van der Linden S : Combatting climate change misinformation: evidence for longevity of inoculation and consensus messaging effects. *J Environ Psychol.* 2020;70: 101455. 10.1016/j.jenvp.2020.101455

[ref-101] Malena-Chan R : A narrative model for exploring climate change engagement among young community leaders. *Health Promot Chronic Dis Prev Can.* 2019;39(4):157–166. 10.24095/hpcdp.39.4.07 31021067 PMC6553576

[ref-102] Malerba F : Sectoral systems of innovation: concepts, issues and analyses of six major sectors in Europe.Cambridge University Press,2004. 10.1017/CBO9780511493270

[ref-103] Marlow T Miller S Roberts JT : Bots and online climate discourses: Twitter discourse on President Trump’s announcement of U.S. withdrawal from the Paris Agreement. *Clim Policy.* 2021;21(6):765–777. 10.1080/14693062.2020.1870098

[ref-104] Martín Serrano M : La epistemología de la dialéctica social. *Revista Española De La Opinión Pública.* 1977; (47):57–76. Reference Source

[ref-105] Martín Serrano M : La producción social de la comunicación. *Signo Y Pensamiento.* 1986;5(9):47–57. Reference Source

[ref-106] McBeth MK Lybecker DL Sargent JM : Narrative empathy: *a narrative policy framework study of working-class climate change narratives and narrators*. *World Affairs.* 2022;185(3):471–499. 10.1177/00438200221107018

[ref-107] McCombs ME Shaw DL : The agenda-setting function of mass media. *Public Opin Q.* 1972;36(2):176–187. 10.1086/267990

[ref-180] McCright AM Dunlap RE : Anti-reflexivity. *Theory, Culture & Society.* 2010;27(2–3). 10.1177/0263276409356001

[ref-108] McCright AM Dunlap RE Marquart-Pyatt ST : Political ideology and views about climate change in the European Union. *Env Polit.* 2015;25(2):338–358. 10.1080/09644016.2015.1090371

[ref-109] McDonough W Braungart M : Cradle to cradle: remaking the way we make things.North Point Press,2002. Reference Source

[ref-110] McGuire WJ : The effectiveness of supportive and refutational defenses in immunizing and restoring beliefs against persuasion. *Sociometry.* 1961a;24(2):184–197. 10.2307/2786067

[ref-111] McGuire WJ : Resistance to persuasion conferred by active and passive prior refutation of the same and alternative counterarguments. *J Abnorm Soc Psychol.* 1961b;63(2):326–332. 10.1037/h0048344

[ref-112] McGuire WJ : Some contemporary approaches. *Adv Exp Soc Psychol.* 1964;1(1):91–229. 10.1016/S0065-2601(08)60052-0

[ref-113] McKibben W : The end of nature.Randhom house,1989. Reference Source

[ref-114] McQuaid K Vanderbeck RM Valentine G : Urban climate change, livelihood vulnerability and narratives of generational responsibility in Jinja, Uganda. *Africa.* 2018;88(1):11–37. 10.1017/S0001972017000547

[ref-115] Moscato D Valencia R : Global narratives of ecological modernization: the construction of climate change op-eds in China Daily and the New York Times. *J Commun Inq.* 2023. 10.1177/01968599231151969

[ref-116] Mulvey K Shulman S Anderson D : The climate deception dossiers: internal fossil fuel industry memos reveal decades of corporate disinformation.Union of Concerned Scientists.2015. Reference Source

[ref-117] Naguimbing-Manlulu MJ : Climate change narratives in Philippine print news media. *Media Asia.* 2021;48(3):190–206. 10.1080/01296612.2021.1944541

[ref-118] Nordensvard J Ketola M : Populism as an act of storytelling: analyzing the climate change narratives of Donald Trump and Greta Thunberg as populist truth-tellers. *Env Polit.* 2022;31(5):861–882. 10.1080/09644016.2021.1996818

[ref-119] Ogra A : Situating climate change narrative for conceptualizing adaptation strategies: a case study of coffee growers in South India. *Reg Environ Change.* 2022;22(2): 72. 10.1007/s10113-022-01919-x

[ref-120] O’Harrow R Jr : A two-decade crusade by conservative charities fueled Trump’s exit from Paris climate accord. *The Washington Post.* 2017. Reference Source

[ref-121] Olmeda TM : Del negacionismo climático al obstruccionismo: el argumentario de la inacción y su amplificación en YouTube. *Dilemata.* 2022;38:119–134. Reference Source

[ref-122] Ong EK Glantz SA : Tobacco industry efforts subverting International Agency for Research on Cancer's second-hand smoke study. *Lancet.* 2000;355(9211):1253–1259. 10.1016/S0140-6736(00)02098-5 10770318

[ref-123] Oreskes N : My Facts Are Better Than Your Facts: Spreading Good News about Global Warming.In: P. Howlett, M. S. Morgan (Eds.), *How well do facts travel?.* Cambridge University Press,2011;136–167.

[ref-124] Oreskes N Conway EM : Merchant of doubt: How a handful of scientists obscured the truth on issues from tobacco smoke to global warming.Bloomsbury Publishing USA;2011. Reference Source

[ref-125] Page MJ McKenzie JE Bossuyt PM : The PRISMA 2020 statement: An updated guideline for reporting systematic reviews. *Int J Surg.* 2021;88: 105906. 10.1016/j.ijsu.2021.105906 33789826

[ref-126] Palm R Lewis GB Feng B : What causes people to change their opinion about climate change? *Ann Am Assoc Geogr.* 2017;107(4):883–896. 10.1080/24694452.2016.1270193

[ref-127] Porter E Wood TJ Bahador BV : Can presidential misinformation on climate change be corrected? Evidence from Internet and phone experiments. *Research & Politics.* 2019;6(3):1–10. 10.1177/2053168019864784

[ref-128] Powell A : Tracing big oil’s PR war to delay action on climate change. *The Harvard Gazette.* 2021. Reference Source

[ref-129] Reay D : Climate science: denialism deciphered. *Nature.* 2016;538(7623):34–35. 10.1038/538034a

[ref-130] Ricoeur P : Ideología y Utopía.Gedisa,1994.

[ref-131] Ringsmose J Børgesen BK : Shaping public attitudes towards the deployment of military power: NATO, Afghanistan and the use of strategic narratives. *European Security.* 2011;20(4):505–528. 10.1080/09662839.2011.617368

[ref-132] Roe MJ Shapira R : The power of the narrative in corporate Lawmaking. *Harv Bus L.* 2021;11(233). Reference Source

[ref-133] Romero-Luis J Carbonell-Alcocer A Gertrudix M : What is the maturity level of Circular Economy and bioenergy research addressed from education and communication? a systematic Literature Review and epistemological perspectives. *J Clean Prod.* 2021;322: 129007. 10.1016/j.jclepro.2021.129007

[ref-134] Ruiu ML Ragnedda M : Use of science in British newspapers’ narratives of climate change. *Studies in Communication Sciences.* 2021;21(2):247–266. 10.24434/j.scoms.2021.02.004

[ref-135] Sambrook K Konstantinidis E Russell S : The role of personal experience and prior beliefs in shaping climate change perceptions: a narrative review. * Front Psychol.* 2021;12: 669911. 10.3389/fpsyg.2021.669911 34276492 PMC8284052

[ref-136] Schmid-Petri H Bürger M : The effect of misinformation and inoculation: replication of an experiment on the effect of false experts in the context of climate change communication. *Public Underst Sci.* 2022;31(2):152–167. 10.1177/09636625211024550 34549661 PMC8814941

[ref-137] Segal M : The missing climate change narrative. *South Atl Q.* 2017;116(1):121–128. 10.1215/00382876-3749370

[ref-138] SEI, Climate Analytics, E3G *et al.* : Phasing down or phasing up? Top fossil fuel producers plan even more extraction despite climate promises. *Production Gap report.* 2023. 10.51414/sei2023.050

[ref-139] Sherif M Hovland CI : Social judgment: Assimilation and contrast effects in communication and attitude change.Yale University Press,1961. Reference Source

[ref-140] Silva HM : Information and misinformation about climate change: lessons from Brazil. *Ethics Sci Environ Polit.* 2022;22:51–56. 10.3354/esep00201

[ref-141] Smith FL : Europe, energy & the environment: the case against carbon taxes.CEI: Competitive Enterprise Institute,1992.

[ref-143] Soon C Goh S : Fake news, false information and more: countering human biases.Institute of Policy Studies,2018. 10.25818/t406-zy24

[ref-144] Stolley PD : Reviewed work: the heat is on: the high stakes battle over earth's threatened climate Ross Gelbspan. *J Public Health Policy.* 1999;20(2):235–237. 10.2307/3343215

[ref-145] Supran G : Fueling their own climate narrative. *Science.* 2021;374(6568):702. 10.1126/science.abm3434 34735252

[ref-146] Supran G Oreskes N : Assessing exxonmobil’s climate change communications (1977–2014). *Environ Res Lett.* 2017;12(8): 084019. 10.1088/1748-9326/aa815f

[ref-147] Supran G Oreskes N : Addendum to ‘Assessing ExxonMobil’s climate change communications (1977–2014)’ Supran and Oreskes (2017 Environ. Res. Lett. 12 084019). *Environ Res Lett.* 2020:15(11): 119401. 10.1088/1748-9326/ab89d5

[ref-148] The National Intelligence Council: Global trends 2040. a more contested world.The National Intelligence Council, USA,2021a. Reference Source

[ref-149] The National Intelligence Council: National Intelligence Estimate. Climate change and international responses increasing challenges to US national security through 2040.2021b. Reference Source

[ref-150] Tomkins SS : Affect, imagery, consciousness: the positive affects. 1962;1. 10.1037/14351-000

[ref-151] Treen KMDI Williams HT O'Neill SJ : Online misinformation about climate change. *Wiley Interdiscip Rev Clim Change.* 2020;11(5): e665. 10.1002/wcc.665

[ref-152] Trudel R : Sustainable consumer behavior. *Consumer Psychology Review.* 2019;2(1):85–96. 10.1002/arcp.1045

[ref-154] Turner JC Hogg MA Oakes PJ : Rediscovering the social group: a self-categorization theory.Basil Blackwell,1987. Reference Source

[ref-155] Turrentine J : The “Big Tent” of people who want climate action just keeps getting bigger. 2019. Reference Source

[ref-156] United Nations: "We didn’t turn the page on the fossil fuel era but this outcome is the beginning of the end": UN climate change executive secretary at COP 28 closing.United Nations. Climate Change,2023. Reference Source

[ref-157] Van der Linden S Leiserowitz A Rosenthal S : Inoculating the public against misinformation about climate change. *Glob Chall.* 2017;1(2): 1600008. 10.1002/gch2.201600008 31565263 PMC6607159

[ref-158] Viotti EB : National learning systems: a new approach on technological change in late industrializing economies and evidences from the cases of Brazil and South Korea. *Technol Forecast Soc Change.* 2002;69(7):653–680. 10.1016/S0040-1625(01)00167-6

[ref-159] Vu HT Baines A Nguyen N : Fact-checking climate change: an analysis of claims and verification practices by fact-checkers in four countries. *Journalism & Mass Communication Quarterly.* 2022;100(2):286–307. 10.1177/10776990221138058

[ref-160] Walther JB Parks MR : Cues filtered out cues filtered in: computer-mediated communication and relationships.In: G. R. Miller (Ed.) *Handbook of interpersonal communication.* Sage,2002;529–563. Reference Source

[ref-161] Walton C : Spies, election meddling, and disinformation: past and present. *The Brown Journal of World Affairs.* 2019;XXVI(1):107–124. Reference Source

[ref-162] Willis R : Too hot to handle?: the democratic challenge of climate change.Policy Press,2020.

[ref-163] Williams MN Bond CM : A preregistered replication of “Inoculating the public against misinformation about climate change”. *J Environ Psychol.* 2020;70: 101456. 10.1016/j.jenvp.2020.101456

[ref-164] Wolff L Taddicken M : *Disinforming the unbiased:* How online users experience and cope with dissonance after climate change disinformation exposure. *New Media & Society.* 2022; 14614448221090194. 10.1177/14614448221090194 1090194

[ref-165] Zhou Y Shen L : Confirmation bias and the persistence of misinformation on climate change. *Commun Res.* 2021;49(4):500–523. 10.1177/00936502211028049

