# Supplementary material for: Disinformation as an obstructionist strategy in climate change mitigation: a review of the scientific literature for a systemic understanding of the phenomenon
Source: Open Res Eur. 2024 Sep 24;4:169. Originally published 2024 Aug 6. [Version 2] doi: 10.12688/openreseurope.18180.2 (PMC11467642; doi:10.12688/openreseurope.18180.2)
Supplement: Peer review history

# Reviewer report: Reviewer response for version 2

**DOI:** 10.21956/openreseurope.20079.r44559

**Published:** 2024-12-18

**Authors:** John Cook

**Affiliations:** 1 The University of Melbourne, Melbourne, Victoria, Australia

Thanks for the diligent Reponses. I just have one further comment - the amended H1 doesn't address my initial concern which was the phrase "deeper and more effective" - it needs to state compared to what? Studies that don't address "financial, economic, and political roots"? And if its not possible to make this comparison (which is fine if it's not, it might be too complex a question to robustly address), then H1 would be better reframed as a research question. Note also the typo: "tat" should be "that"

Are sufficient details of the methods and analysis provided to allow replication by others?

Yes

Are the rationale for, and objectives of, the Systematic Review clearly stated?

Yes

Is the statistical analysis and its interpretation appropriate?

Yes

Are the conclusions drawn adequately supported by the results presented in the review?

Partly

Reviewer Expertise:

My area of expertise is climate misinformation, including psychological experiments testing corrections, development and testing of inoculation interventions, and computer science research into automatic detection and generative debunking of climate misinformation.

I confirm that I have read this submission and believe that I have an appropriate level of expertise to confirm that it is of an acceptable scientific standard.

# Reviewer report: Reviewer response for version 2

**DOI:** 10.21956/openreseurope.20079.r44781

**Published:** 2024-10-22

**Authors:** Ana Delicado, Jussara Rowland

**Affiliations:** 1 Instituto de Ciências Sociais da Universidade de Lisboa, Lisbon, Portugal

The article provides a comprehensive literature review on climate disinformation and obstructionist strategies related to climate change mitigation. It has undergone several rounds of review, and overall, the study is based on a sound methodology, with clear and well-supported conclusions based on the results presented.

 However, I have a concern regarding the treatment of the concepts of "disinformation" and "misinformation." In response to previous reviewers, the authors introduced a distinction between these terms, referencing definitions from the European Community. They also note that there is a lack of conceptual clarity in the existing literature on these terms. Specifically, they state:

 “On the other hand, when reviewing the scientific literature, we can see that time and again an extensive and undifferentiated use of concepts is made, leading to greater confusion about the nature of the phenomenon at a systemic level. This can be observed with the terms disinformation and misinformation, which are often used indiscriminately without referring to or attributing the malicious nature of disinformation.”

 Despite making this critique, the article itself tends to use these terms interchangeably. At times, the distinction between "disinformation" and "misinformation" seems well-justified and aligns with what is being stated in the article. However, in other instances, the usage lacks clarity. This inconsistency is noticeable from the very beginning. While the title highlights disinformation, the abstract begins with:

 “This study examines the scientific misinformation about climate change, in particular obstructionist strategies”

 While this does not significantly affect the overall analysis or conclusions, greater conceptual clarity would strengthen the paper. I recommend that the authors either review and adjust the usage of these terms or introduce a statement explaining how the two concepts are used in the context of the article. This would resolve potential confusion and enhance the paper's conceptual rigor.

 On a minor note, there is a misplaced parenthesis in "based on an earlier analysis by ( Ong & Glantz, 2000)".

Are sufficient details of the methods and analysis provided to allow replication by others?

Yes

Are the rationale for, and objectives of, the Systematic Review clearly stated?

Yes

Is the statistical analysis and its interpretation appropriate?

Yes

Are the conclusions drawn adequately supported by the results presented in the review?

Yes

Reviewer Expertise:

Sociology, Science and Technology Studies. Science Communication

We confirm that we have read this submission and believe that we have an appropriate level of expertise to confirm that it is of an acceptable scientific standard.

# Reviewer report: Reviewer response for version 2

**DOI:** 10.21956/openreseurope.20079.r44778

**Published:** 2024-10-10

**Authors:** David Vicente Torrico, Damián Moreno Prado

**Affiliations:** 1 University of Valladolid, Valladolid, Castilla y León, Spain

The research titled “Disinformation as an obstructionist strategy in climate change mitigation: a review of the scientific literature for a systemic understanding of the phenomenon” presents a detailed study of the publications that address a topic of special relevance and relevance, such as disinformation about climate change and the social impact it produces. As noted in previous reviews, the article stands out for its methodological approach, theoretical foundation and justification of the findings. In this sense, the authors have appropriately incorporated most of the observations made after the first review: distinction between disinformation and misinformation; definition of critical media literacy; clarifications, justifications and terminological changes at different points of the work.

 On the contrary, we believe that the paragraph dedicated to Hahn's criticism of Al Gore's book should be revised again, since the wording continues to be confusing. Specifically:

 - “He portrays the denier lobbies…: By “he” do you mean Hahn or Gore?

 - “Who are portrayed are romantically”…: Is it possible that about an “are”?

 - ... with disinformation” / are romantically”…: There are two closing quotes in a row

 To avoid recurring references to the work of Almiron & Moreno (2022), it is suggested that you consult two publications whose content is related to this topic:

 - Heras, F..et.al., 2018 (Ref 2)

 - Abellán López , M. Á.et.al., ( 2021 ) (Ref 1)

 Additionally, the following observations are made:

 - P.4 => It would be desirable to use shorter sentences to avoid repetition of structures in the fifth paragraph (and/others)

 - P.5 => “The Heat is on / “the organization… => There are two opening quotes in a row

 - P.7 => In objectives it would be advisable to remember the time frame of the study

 - Q.7 => In H1 there is a typo: tat instead of that

 - P.11 => Tables 3 and 4 contain three terms marked with an * whose meaning is not clarified in the text

 - P.12 => The last paragraph expresses the present tense (are) and not the past tense (were)

 - P.13 => “The following diagram…” does not refer to any subsequent image, but rather a previous one

 - The arrangement of images can be confusing when they are far from the text

 - The tables and images help to understand the methodological design, but they would also be useful to provide a quantitative view of the results of the analysis, which generally remain on a qualitative descriptive level.

 - P.17 => The use of synonyms is recommended in “research findings, finding results that qualify or challenge the van der Linden et al. (2017) findings”.

 - P.18 => In the second paragraph of Discussion “that that” appears

 - P.19 => The use of synonyms is recommended in the second conclusion “diversity and diversity”

 - P.20 => The second paragraphs of each conclusion appear tabulated differently. If it is not an editorial issue, it is recommended that you review it.

Are sufficient details of the methods and analysis provided to allow replication by others?

Yes

Are the rationale for, and objectives of, the Systematic Review clearly stated?

Yes

Is the statistical analysis and its interpretation appropriate?

Yes

Are the conclusions drawn adequately supported by the results presented in the review?

Yes

Reviewer Expertise:

Our lines of research address scientific communication in a broad sense, with some approaches to the field of obstructionism and climate disinformation.

We confirm that we have read this submission and believe that we have an appropriate level of expertise to confirm that it is of an acceptable scientific standard.

## References

[1] : El cambio climático: negacionismo, escepticismo y desinformación. *Tabula Rasa* .2021; 10.25058/20112742.n37.13 10.25058/20112742.n37.13

[2] : Responses to climate denialism. *https://www.fuhem.es/papeles_articulo/respuestas-ante-el-negacionismo-climatico/* .2018;

# Reviewer report: Reviewer response for version 2

**DOI:** 10.21956/openreseurope.20079.r44558

**Published:** 2024-09-25

**Authors:** Antonio Lopez

**Affiliations:** 1 John Cabot University, Rome, Italy

I approve, but please fix the formatting of this reference: López A: Gaslighting: fake climate news and big carbon’s network of Denial. 2023; 159–177.

 This is the complete reference: López, A. (2023). Gaslighting: Fake Climate News and Big Carbon’s Network of Denial. In: Fowler-Watt, K., McDougall, J. (eds) The Palgrave Handbook of Media Misinformation. Palgrave Macmillan. https://doi.org/10.1007/978-3-031-11976-7_11

Are sufficient details of the methods and analysis provided to allow replication by others?

Yes

Are the rationale for, and objectives of, the Systematic Review clearly stated?

Yes

Is the statistical analysis and its interpretation appropriate?

Yes

Are the conclusions drawn adequately supported by the results presented in the review?

Yes

Reviewer Expertise:

I'm not an expert in the PRISMA 2020 model, but I have studied quite extensively the history of media and climate disinformation, and I am an expert in media education,

I confirm that I have read this submission and believe that I have an appropriate level of expertise to confirm that it is of an acceptable scientific standard.

## References

[1] : Ecological features facilitating spread of alien plants along Mediterranean mountain roads. *Biol Invasions* .2024;26(11) : 10.1007/s10530-024-03418-y 3879-3899 10.1007/s10530-024-03418-y 39324107 PMC11420372

# Reviewer report: Reviewer response for version 1

**DOI:** 10.21956/openreseurope.19649.r43280

**Published:** 2024-09-10

**Authors:** John Cook

**Affiliations:** 1 The University of Melbourne, Melbourne, Victoria, Australia

This study conducted a systematic literature review of papers about climate obstruction, finding an emphasis on immunizing and fact-checking but few studies on the organizational structures supporting disinformation.

 Overall, I find this is an interesting study that makes a useful contribution. The point about the need for more systemic approaches to disinformation is well made. However, I find the hypotheses and their partial confirmation unsatisfying. It seems hand-wavy and ill-defined on whether the literature review answers the hypotheses in a robust way. The hypotheses use phrases like “provides a deeper”, “better able to”. Deeper or better compared to what? How does the literature review determine whether certain studies are better than others? In the discussion section, justification of partial confirmation needs to be explained better. Or if it’s not possible to rigorously confirm or disconfirm hypotheses of this nature, perhaps they are better off framed as research questions.

 I have some minor comments on the text as well:

**Introduction:** You state that "Systematic disinformation... has a profound impact on decision-making, public trust and the effectiveness of policies and the actions they promote". What's the citation for this? 

 Just a comment: the paper leans heavily on Almiron & Moreno (2022). Excellent to offer more perspective from non-WEIRD countries but pity this paper isn’t available in English as I’d like to read it!

 On page 5: what is the “E)” (after the five areas of corrupting the scientific field)?

 Page 6: I find the paragraph about Al Gore confusing. After reading it several times, I think you’re talking about a denialist review of Al Gore’s book but it’s not clear – I suggest clarifying the text, perhaps by mentioning who wrote the review or more context around the review early in the paragraph.

 Page 6: The paragraph about the Chris Horner book – I’d rewrite the sentence that uses the word “which” twice to be more readable.

 Page 6: Is the neoconservative line “inescapably” linked or “inextricably” linked to Trump?

 Page 6: The transition to talking about the Intelligence Community report is abrupt, doesn’t really seem to fit in the flow of the text. Seems that paragraph could be deleted with no disruption to the manuscript.

 Page 9: The tense shifts from past tense to present tense halfway through describing the methodology. Is this intentional?

 Page 11: Use of “Ambito”, do you want this to be English?

 Page 12: The tense now shifts to future tense. Recommend consistency of past tense throughout the methodology section.

 Page 13: Should the subtitle “Identification of studies via other methods” be formatted like other subheadings?

 Page 16: I question the characterization of the phrase “Research by McBeth,

 Lybecker and Sargent (2022), in line with the results of Benegal and Scrugg (2018), questions the predictive power of political ideology for climate attitudes.” The citations don’t question the predictive power of ideology but show that certain contexts can reduce the influence of ideology in causing rejection of climate messages. Political ideology is still predictive but can be mitigated. This characterization is brought up again on page 17/18 when summarising Benegal and Scruggs (2018).

 Page 18: I’d reword “The authors saw positive effects in the use of vaccination messages” to “inoculation messages” as inoculation messages are not called vaccination messages in the psychological literature. The term vaccination or vaccine is used again several times in the same and subsequent paragraphs and should be changed to inoculation.

Are sufficient details of the methods and analysis provided to allow replication by others?

Yes

Are the rationale for, and objectives of, the Systematic Review clearly stated?

Yes

Is the statistical analysis and its interpretation appropriate?

Yes

Are the conclusions drawn adequately supported by the results presented in the review?

Partly

Reviewer Expertise:

My area of expertise is climate misinformation, including psychological experiments testing corrections, development and testing of inoculation interventions, and computer science research into automatic detection and generative debunking of climate misinformation.

I confirm that I have read this submission and believe that I have an appropriate level of expertise to confirm that it is of an acceptable scientific standard, however I have significant reservations, as outlined above.

# Response

**Published:** 2024-09-15

**Authors:** Manuel Gertrudix

**Affiliations:** University Rey Juan Carlos, Spain

Thank you for your detailed feedback. In response to your concerns about the hypotheses, we have revised them to improve their precision and eliminate vague wording. For example, H1 now emphasises "identifying more precisely the dynamics that sustain disinformation" and "developing targeted interventions"," while H2 focuses on "quantitative and qualitative methods" and developing "operational strategies"." H3 has been refined to emphasise a "holistic understanding" of the financial, economic and political factors underlying climate disinformation. These adjustments have also led to corresponding revisions in the discussion to ensure coherence with the updated hypotheses. Regarding the citation on the impact of systematic disinformation on decision-making and public trust, a reference to Abudu et al. (2023) has been added. We have also addressed formatting and clarity issues in the text. For example, we clarified the paragraph about Al Gore’s book by explicitly mentioning the author of the review, Stephen Hahn, and reworded the paragraph about Chris Horner’s book for better readability. We also replaced "inescapable" with "inextricable" to improve the precision of the language, and revised the section on political rhetoric to better connect it to the national security implications of climate change. In addition, we have corrected inconsistencies in tense in the methodology section, ensuring consistent use of the past tense and replacing "Ámbito" with "Area" to ensure consistent terminology. We also reformatted the subtitle "Identification of studies using other methods" to match the style of the manuscript’s subheadings. Finally, the term "vaccination messages" has been replaced with "vaccination messages" throughout the manuscript to reflect correct psychological terminology.

# Reviewer report: Reviewer response for version 1

**DOI:** 10.21956/openreseurope.19649.r42882

**Published:** 2024-09-10

**Authors:** Antonio Lopez

**Affiliations:** 1 John Cabot University, Rome, Italy

The article titled "Disinformation as an Obstructionist Strategy in Climate Change Mitigation" provides a comprehensive review of the scientific literature on climate change misinformation, aiming to understand the systemic nature of disinformation and its impact on public perception and policy. The study clearly states its rationale and objectives and the methodology is detailed enough to allow for replication (it employs a systematic literature review using the PRISMA 2020 model). The statistical analysis is appropriate, and the conclusions are well-supported by the results, emphasizing the need for global and comparative research to combat misinformation effectively. The systematic review identifies several key findings regarding the spread and impact of climate disinformation. Firstly, it highlights that technological solutions like fact-checking and misinformation immunization are prevalent, yet there is a lack of in-depth research on the operational structures that sustain systematic disinformation. The review underscores the necessity for more comprehensive and globally coordinated studies, emphasizing the financial, economic, and political roots of disinformation. It calls for better-funded research that spans multiple countries to effectively address the complex web of disinformation. I fully concur with its main findings and was pleased to see them in the discussion section, for one of the main things I identified while reading the reviewed studies is how few of them addressed the political economy of media and technology platforms. I was very happy to see that the authors also observed this. The review does not capture the work of critical media literacy, which has addressed this issue, but there are few studies that address this (ecomedia literacy is one area of media literacy that addresses the crossover between fake news and climate disinformation) ^1,2^. Despite this, I don't think it impacts the overall conclusions. It would be good to have a clear definition at the very beginning that explains the difference between misinformation and disinformation. I would argue that the former is unintentional and the latter is intentional. Beyond this, I believe this is a valuable study that will advance our understanding of how to address climate disinformation. The study's findings are crucial for policymakers, scientists, and the public to develop strategies that promote accurate climate information and support effective climate action.

Are sufficient details of the methods and analysis provided to allow replication by others?

Yes

Are the rationale for, and objectives of, the Systematic Review clearly stated?

Yes

Is the statistical analysis and its interpretation appropriate?

Yes

Are the conclusions drawn adequately supported by the results presented in the review?

Yes

Reviewer Expertise:

I'm not an expert in the PRISMA 2020 model, but I have studied quite extensively the history of media and climate disinformation, and I am an expert in media education,

I confirm that I have read this submission and believe that I have an appropriate level of expertise to confirm that it is of an acceptable scientific standard.

## References

[1] : The Critical Media Literacy Guide. *https://brill.com/display/title/55281?language* .2019;

[2] : Gaslighting: Fake Climate News and Big Carbon's Network of Denial. *https://www.researchgate.net/publication/373993360_Gaslighting_Fake_Climate_News_and_Big_Carbon's_Network_of_Denial* .2023;

# Response

**Published:** 2024-09-15

**Authors:** Manuel Gertrudix

**Affiliations:** University Rey Juan Carlos, Spain

Thank you for your valuable suggestions.

 In response to your feedback, we have included a discussion of critical media literacy (CML) and ecomedia literacy in both the introduction and discussion of the manuscript, which addresses the intersection of fake news and climate disinformation.

 Key works by Kellner & Share (2019) and Lopez (2023) were used to support this analysis.

 We also made a clear distinction between misinformation (unintentional spread of false information) and disinformation (intentional dissemination of false information) at the beginning of the manuscript on your recommendation, using definitions from the European Commission (2018; 2020).
